# The Targeted Impact of Flavones on Obesity-Induced Inflammation and the Potential Synergistic Role in Cancer and the Gut Microbiota

**DOI:** 10.3390/molecules25112477

**Published:** 2020-05-27

**Authors:** Meenakshi Sudhakaran, Andrea I. Doseff

**Affiliations:** 1 Physiology Graduate Program, Michigan State University, East Lansing, MI 48824, USA; sudhaka7@msu.edu; 2 Department of Physiology, Michigan State University, East Lansing, MI 48824, USA; 3 Department of Pharmacology and Toxicology, Michigan State University, East Lansing, MI 48824, USA

**Keywords:** flavones, inflammation, obesity, cancer, microbiome, molecular mechanisms, gene and protein regulatory networks, macrophages, NF-κB, IKKβ, inflammatory cytokines, apoptosis, apigenin, foods for health

## Abstract

Obesity is an inflammatory disease that is approaching pandemic levels, affecting nearly 30% of the world’s total population. Obesity increases the risk of diabetes, cardiovascular disorders, and cancer, consequentially impacting the quality of life and imposing a serious socioeconomic burden. Hence, reducing obesity and related life-threatening conditions has become a paramount health challenge. The chronic systemic inflammation characteristic of obesity promotes adipose tissue remodeling and metabolic changes. Macrophages, the major culprits in obesity-induced inflammation, contribute to sustaining a dysregulated immune function, which creates a vicious adipocyte–macrophage crosstalk, leading to insulin resistance and metabolic disorders. Therefore, targeting regulatory inflammatory pathways has attracted great attention to overcome obesity and its related conditions. However, the lack of clinical efficacy and the undesirable side-effects of available therapeutic options for obesity provide compelling reasons for the need to identify additional approaches for the prevention and treatment of obesity-induced inflammation. Plant-based active metabolites or nutraceuticals and diets with an increased content of these compounds are emerging as subjects of intense scientific investigation, due to their ability to ameliorate inflammatory conditions and offer safe and cost-effective opportunities to improve health. Flavones are a class of flavonoids with anti-obesogenic, anti-inflammatory and anti-carcinogenic properties. Preclinical studies have laid foundations by establishing the potential role of flavones in suppressing adipogenesis, inducing browning, modulating immune responses in the adipose tissues, and hindering obesity-induced inflammation. Nonetheless, the understanding of the molecular mechanisms responsible for the anti-obesogenic activity of flavones remains scarce and requires further investigations. This review recapitulates the molecular aspects of obesity-induced inflammation and the crosstalk between adipocytes and macrophages, while focusing on the current evidence on the health benefits of flavones against obesity and chronic inflammation, which has been positively correlated with an enhanced cancer incidence. We conclude the review by highlighting the areas of research warranting a deeper investigation, with an emphasis on flavones and their potential impact on the crosstalk between adipocytes, the immune system, the gut microbiome, and their role in the regulation of obesity.

## 1. Introduction

The incidence of obesity has ascended steadily in the last ~35 years and is reaching epidemic levels worldwide, inflicting life-threatening conditions and great socioeconomic burden. It is an alarming fact that almost half of the world’s population is obese or overweight. Obesity is a major health concern often correlated with deteriorating life expectancy and increasing risks of several comorbid disorders, such as cardiovascular diseases, hypertension, type-2 diabetes mellitus, non-alcoholic fatty liver disease, steatohepatitis, osteoarthritis, and cancer [1]. The significant increase in obesity within the world’s population prompted the need for identifying novel cost-effective interventions that are capable of controlling obesity with minimal harmful side effects. Obesity and obesity-linked diseases are associated with systemic chronic inflammation that leads to altered adipocyte functions [2]. Aberrant accumulation of macrophages (referred to as adipose tissue associated macrophages or ATMs) in adipose depots and other immune cells are vital contributors to obesity-induced inflammation [3,4]. The discovery that immune cell infiltration increases in adipose tissues of obese individuals has opened a new aspect in the research field and emphasizes the interest of using strategies that target immune cells to overcome the adversities associated with obesity. Thus, elucidating the mechanisms underlying obesity-linked inflammation has been suggested as a potential approach in preventing and battling obesity. High fat diets (HFD) induced harmful changes in the gut microbiome, leading to inflammation and systemic metabolic dysregulation [5]. Therefore, regulating the gut microbiome through the use of healthier diets could impact prevention and treatment of obesity.

Flavonoids are a large class of bioactive dietary nutraceuticals derived from the phenolic metabolism, which is widely distributed in plants and represents the important nutritional components of our diet [6]. Flavonoids, with more than 7000 identified, so far, have a myriad of health-promoting effects, owing to their potent antioxidant, anti-inflammatory, anti-carcinogenic, and immuno-modularity properties [7,8]. Due to these benefits, flavonoids are attracting great interest in the treatment and prevention of chronic inflammatory diseases. Emerging evidence suggests that the intake of flavonoid-rich diets exerts an inverse correlation with obesity and related inflammation [9,10]. Interestingly, recent studies showed that flavonoids can alter the gut microbiota ecosystem, reducing systemic inflammation [11]. Here, we reviewed the mechanistic aspects of obesity-induced inflammation, as well as the current knowledge on the role of dietary flavones, a subclass of flavonoids, and the molecular mechanisms that are involved in regulating obesity-induced inflammation and related diseases, such as cancer. We also highlight the potential beneficial effects of flavones on the relation between gut microbiota, immune and adipocyte homeostasis, and their impact on controlling and treating obesity.

## 2. Obesity-Induced Inflammation and Its Impact on Health

### 2.1. Obesity

Obesity is defined as an increase in body mass fat, resulting in excessive calorie consumption associated with a high incidence in the development of cardiovascular disease, metabolic dysfunction, diabetes, liver damage, and even cancer. Obesity is characterized by the presence of low and systemic chronic inflammation, which leads to dysregulated adipocyte function, promoting hormonal changes that alter the regulation of food consumption. In mammals, adipose tissue is classified into white adipose tissue (WAT) and brown adipose tissue (BAT). WAT functions as a reservoir of triglycerides from which free fatty acids (FFAs) are released to fuel the energy demands. BAT is considered to be a lipid reserve for cold-induced adaptive thermogenesis and is characterized by an increased mitochondrial count, lipolysis, and expression of uncoupling protein-1 (UCP-1), a key protein involved in the regulation of energy expenditure and protection against oxidative stress [12]. WAT is divided into two main depots, the subcutaneous adipose tissue (SAT) found under the skin, and the visceral adipose tissue (VAT) located around the internal organs. Among these depots, two types of thermogenic adipocytes are known to exist—classical brown and beige, which have disparate developmental and anatomical characteristics. The classical brown adipocytes found in the BAT have an embryonic origin, whereas the inducible thermogenic beige adipocytes exist in WAT and are derived either through transdifferentiation of WAT or from beige adipocyte precursor cells expressing platelet-derived growth factor receptor (PDGFR)-α [13]. This occasional development of beige adipocytes is referred to as the browning of WAT and was first identified in rodents, however, recent findings suggest the presence of beige adipocytes in humans as well. Browning is associated with resistance to HFD-induced obesity. In obese individuals, the conversion of triglycerides into FFAs through lipolytic enzymes such as adipose triglyceride lipase (ATGL) and hormone-sensitive lipase (HSL), and the subsequent FFA β-oxidation are impaired, consequently affecting the browning of adipose tissues [14]. Adipose tissue is also recognized as an endocrine organ that regulates systemic energy homeostasis, releasing a repertoire of cytokines (referred to as adipokines), hormones, and lipokines [15,16]. Leptin, an adipokine secreted during food intake, plays a key role in maintaining metabolic balance by suppressing appetite. Leptin levels are highly upregulated in obesity, but obese individuals become “leptin resistant” by losing their ability to control food ingestion, despite the presence of high levels of leptin [17]. The adipokine adiponectin has anti-obesogenic functions and regulates glucose levels and lipid oxidation [18]. Interestingly, recent studies involving single cell sequencing, and metabolomic and proteomic analyses of human mesenchymal progenitors and WAT, identified adipocyte progenitors that developed into adipocyte subsets with distinct metabolic and endocrine functions [19,20]. These findings highlight the cellular heterogeneity of adipose tissues and the need to gain a better understanding of the adipocyte populations, its precursors and the regulatory mechanisms that define their role in obesity.

Adipogenesis is a multi-step process that involves the development of a multipotent mesenchymal stem cell into a precursor preadipocyte, which then further differentiates into a mature lipid-laden adipocyte [21]. In the mouse cell line 3T3-L1, a broadly used model of adipogenesis, mitotic clonal expansion (MEC) involving multiple cell divisions, precedes the terminal differentiation [22]. Adipogenesis is regulated by a cascade of transcription factors, including peroxisome proliferator-activated receptor gamma (PPARγ), CAAT/enhancer-binding proteins (C/EBP), and sterol regulatory element binding protein isoform (SREBP)-1c, which induce temporal changes of adipogenesis-regulatory genes [23,24,25]. A recent study using a single molecule 5′ cap analysis of gene expression (CAGE) revealed dynamic patterns of gene expression profiles during adipocyte differentiation, in which the early stage involved an increase in genes related to structural remodeling and cell division, whereas genes in the later differentiation state were involved in the regulation of lipid metabolism and energy homeostasis characteristic to WAT [26]. Human transcriptome analyses reported several adipocyte-specific genes, such as leptin, adiponectin, fatty acid binding protein (FABP)-4 and ATGL, to be highly expressed in mature adipocytes [27].

**Figure 1:**
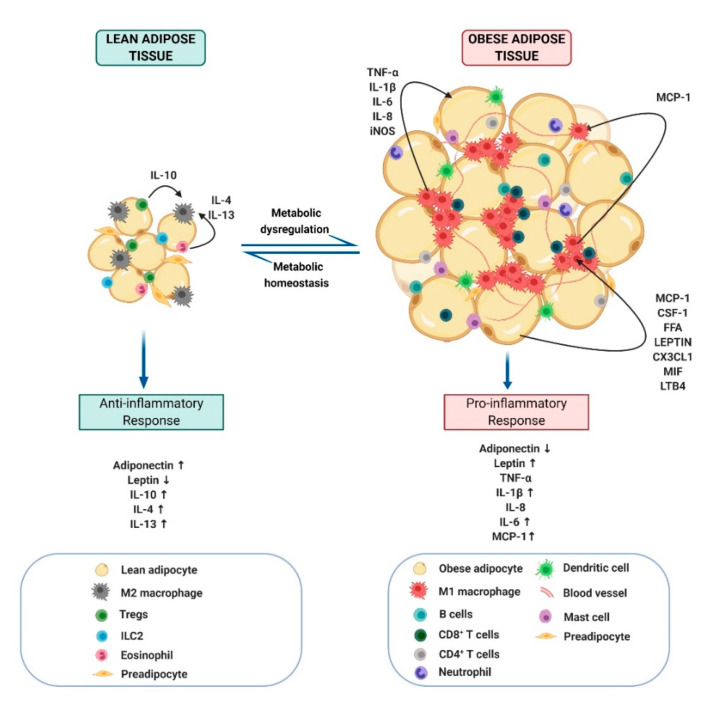
Schematic representation of the cellular dynamics of adipose tissue associated with obesity. As obesity develops, hypertrophic adipocytes and changes in immune cell populations contribute to the development of a chronic inflammatory adipose microenvironment that leads to metabolic dysregulation.

In obesity, an imbalance in energy homeostasis causes adipose tissue remodeling, including adipocyte enlargement (hypertrophy) and an increase in numbers (hyperplasia) [28]. The adipose tissue constitutes adipocytes and stroma, which includes endothelial cells, pericytes, adipose stem cells, and immune cells (Figure 1). In lean conditions, macrophages, the predominant immune cell population accounting for 10% of all cells in the adipose tissue, are found in an alternatively activated M2 state, characterized by expressing CD206^+^ CD163^+^ CD301^+^ surface receptors and are sparsely distributed [3]. Loss of M2 macrophages resulted in increased weight gain in myeloid-specific PPAR delta (δ) ablated mice fed with HFD, reflecting on the relevance of M2 ATMs in mitigating obesity [29]. In obese conditions, there is a significant increase in the number of ATMs, which are mainly found as classically activated M1 phenotype expressing CD11c^+^ CD86^+^ surface proteins responsible for promoting inflammatory conditions [30,31]. Notably, the main mechanism contributing to the increase of M1 phenotype is the recruitment of inflammatory monocytes (characterized by the presence of CCR2^++^ CX3CR1^low^ Ly6C^high^ CD11b^+^ surface proteins) from the circulation, which on entering the adipose tissue differentiate into M1 ATMs [32]. The adipocyte secreted chemokine monocyte chemoattractant protein (MCP)-1 is known to be a key player in recruiting inflammatory monocytes to the adipose tissue [3,33]. Transgenic mice overexpressing MCP-1 showed an increased number of infiltrated macrophages in the adipose tissue, supporting the relevance of MCP-1 [34]. The recruitment of monocytes also requires CD11b integrin, as demonstrated using CD11b-deficient HFD-fed mice [35]. Several other adipocyte-induced chemokines such as colony stimulating factor (CSF)-1, C-X3-C motif ligand (CX3CL)-1, leukotriene B4, and macrophage migration inhibitory factor (MIF) also promoted macrophage infiltration [3,36,37,38]. Additionally, it was suggested that the proliferation of resident M2 ATMs might also contribute to an increase in the ATM population at the early stages of obesity [39,40]. Recent findings revealed the presence of a distinct pool of proinflammatory metabolically activated macrophages (MMe), which were stimulated by palmitate and participated in the trafficking and lysosomal metabolism of lipids, yet, failed to express the typical M1 markers [41,42]. These initial findings suggest a higher ATM heterogeneity than expected and would require further investigation. Other immune cells also contribute to maintaining the metabolic homeostasis in adipose tissue. Tregs and eosinophils aid in the polarization of ATMs into an M2 phenotype, by releasing cytokines such as interleukin (IL)-4, IL-13, and IL-10 [43,44,45,46]. In addition, innate lymphoid cells (ILC)-2 seem to induce adipocyte browning and ameliorate obesity through IL-33 dependent upregulation of UCP-1 [47]. Recent metabolomic and lipidomic profiling studies reported differential metabolite and lipid signatures in human and animal models, revealing a significant increase in glycerol 1-phosphate, glycolic acid, uric acid, polysaturated fatty acids, and fatty acyl chains in obese groups, thereby engendering fatty livers [48,49,50]. These studies highlight the need for the identification of molecules that can be used as an early diagnostic and prognostic marker in obesity-induced inflammation.

### 2.2. Inflammation and Its Link with Obesity

Chronic inflammation is a prolonged and progressive response that is accompanied by an altered immune function, ultimately leading to tissue dysfunction. It plays a key role in the initiation and progression of the pathophysiological alterations that are characteristic of obesity. Although there is an indisputable link between inflammation and obesity, there are still unresolved questions pertaining to its trigger and causative factors. It was hypothesized that inflammation initially originates as a consequence of homeostatic stress due to energy imbalance in the adipocytes [51]. Obesity-induced increase in gut permeability can give rise to circulating intestinal stemmed Gram-negative bacterial lipopolysaccharide (LPS) levels, which provoke inflammatory responses by interacting with toll-like receptor (TLR)-4 in adipocytes and macrophages [52,53]. Additionally, dietary or adipose tissue-derived FFAs binding to TLR2 and TLR4 can trigger the inflammatory signaling pathways [54]. Hypertrophic adipocytes can stimulate local induction of the transcription factor hypoxia-inducing factor (HIF)-1α, as a result of excessive oxygen depletion and decreased perfusion, leading to the upregulation of proinflammatory genes, the FFAs plasma levels, and macrophage infiltration [55,56]. FFAs released from dying adipocytes also exacerbate inflammation by binding to macrophage TLR2/4 receptors triggering the activation of nuclear factor-kappaB (NF-κB) and NOD-like receptor (NLR)P3, through the damage-associated molecular proteins (DAMPs), with subsequent production of inflammatory cytokines [57,58]. Often obese adipose tissues are characterized by the elevated generation of mitochondrial reactive oxygen species (ROS), leading to mitochondrial deregulation, oxidative stress, and inflammation [59]. In response to these stimuli, metabolically dysregulated adipocytes secrete proinflammatory cytokines, such as tumor necrosis factor (TNF)-α, which stimulate the neighboring adipocytes and the endothelial cells to secrete NF-κB regulated MCP-1 and other chemokines, thereby recruiting M1 ATMs [33]. High levels of MCP-1 were also implicated in mediating resident macrophage proliferation [60]. Transgenic and chemically induced mice models lacking macrophages conclusively supported the role of ATMs in promoting obesity-induced inflammation. Ablation of macrophages using clodronate liposomes (CL) treatment resulted in the reduction of HFD-induced adipose tissue inflammation [61]. Moreover, a significant decrease in inflammation and insulin resistance was observed in granulocyte macrophage colony stimulating factor (GM-CSF) knock-out mice fed with HFD, owing to the abatement of C-C motif chemokine receptor (CCR)-2-specific macrophage infiltration in adipose tissues, but had no effect on body weight [62]. Adipocyte-induced netrin-1 promoted the retention of inflammatory macrophages in obese adipose tissues by interacting with specific macrophage receptors [63,64]. The infiltrated M1 ATMs produce significantly large amounts of proinflammatory cytokines such as TNF-α, IL-6, IL-8, and IL-1β and attract more macrophages through MCP-1 secretion, creating a deleterious adipose microenvironment [31,34,65]. M1 macrophages can group around the dying adipocytes to form crown-like structures (CLS), which are indicative of an increase in hypoxia, hypertrophy, and stress [66]. This aberrant inflammatory environment creates further adipocyte dysregulation through increased secretion of leptin and lipolytic genes, and enhanced insulin resistance, eventually leading to adipocyte death.

Additional immune cells including mast cells, neutrophils, dendritic cells, T cells, B cells, and ILCs in the adipose tissue stroma participate in the onset of obesity (Figure 1). Nonetheless, how the interaction between these immune cells initiate and sustain adipose tissue inflammation remains elusive. In obesity, prior to ATM infiltration, there is a rise in the number of lymphocytes in the obese adipose tissues, which affects the sustenance of obesity-induced inflammation but is dispensable for the onset of obesity [4]. CD8^+^ T cells, activated by hypertrophic adipocytes expressing major histocompatibility complex (MHC)-II, enhance ATM filtration and adipose tissue inflammation in HFD-fed mice, as confirmed by the CD8^+^ T cells loss and gain of function in vivo studies [67,68,69]. CD8^+^ T cells were found around CLS along with M1 ATMs in epididymal adipose tissues (EAT). Interestingly, CD4^+^ T cells were identified to exhibit long-lasting obesity memory and induction of body mass regain in a weight gain–loss–regain C57BL/6J model, suggesting the potential role of an immune cell stimulated inflammatory condition in promoting obesity relapse [70]. B cells also stimulated secretion of proinflammatory cytokines, adipocyte hypertrophy and insulin resistance in obese mice. A decrease in systemic inflammation in obese B-cell-deficient mice was correlated with a significant reduction in Tregs, indicating the ability of B cells to regulate the T cell function in hypertrophic adipose tissue [71]. Additionally, B cell filtration in adipose tissues seems to precede other immune cells. B cells were infiltrated into the adipose tissues after 3 weeks of HFD, followed by T cell at week 6, and macrophage infiltration at week 12. Importantly, there was a significant rise in ATMs and natural killer cells (NK) in lymphocyte-deficient Rag2^-/-^ mice, suggesting that T cells and B cells are not essential for the initiation of obesity [72]. Additionally, it remains debatable whether the early lymphocyte accumulation is a protective response rather than a stimulus of inflammatory conditions. Loss and gain of function in HFD-fed and leptin-mutated genetically obese mice revealed that CD4^+^ Foxp3^+^ Tregs secreted anti-inflammatory cytokines like IL-10 and influenced the insulin sensitivity of adipose tissues [44]. Interestingly, single cell RNA-sequencing of SAT, identified crosstalk induced between the adipocytes and the IL-10 secreting immune cells, wherein beige-like metabolically active adipocytes exhibited an enhanced expression of IL-10Rα responsive thermogenic genes [73]. A potential role of mast cells in mediating systemic thermogenesis, macrophage recruitment, and insulin resistance in high cholesterol and HFD-fed mice was reported [74]. Eosinophils-induced IL-4 in WAT was found to promote M2 macrophage polarization [75,76]. Neutrophils promote inflammation in HFD-induced obese mice, through the secretion of proteases, such as elastase [77]. CD11c^+^ CD64^-^ expressing dendritic cells accumulate in the SAT of obese mice and in humans, which promotes the activation of CD4^+^ T-cell polarization and proliferation through Th17-type responses, to trigger inflammation [78,79]. The role of ILCs in obesity remains controversial. ILC2 depletion was reported to decline eosinophils, M2 ATMs, and anti-inflammatory cytokines, such as IL-13 and IL-5 in the VAT, thereby inducing adiposity [80]. On the contrary, recent studies identified that the loss of ILC2 and ILC3 resulted in decreased weight-gain in HFD-fed mice, which was reversed through the adoptive transfer of small intestine ILC2 [81]. The discrepancies in these studies suggest that further investigations to evaluate the role of different immune cells are necessary. Collectively, these findings support the complex roles of innate and adaptive immune cells during the early stages of obesity-induced inflammation, contributing to adipose tissue remodeling.

**Figure 2:**
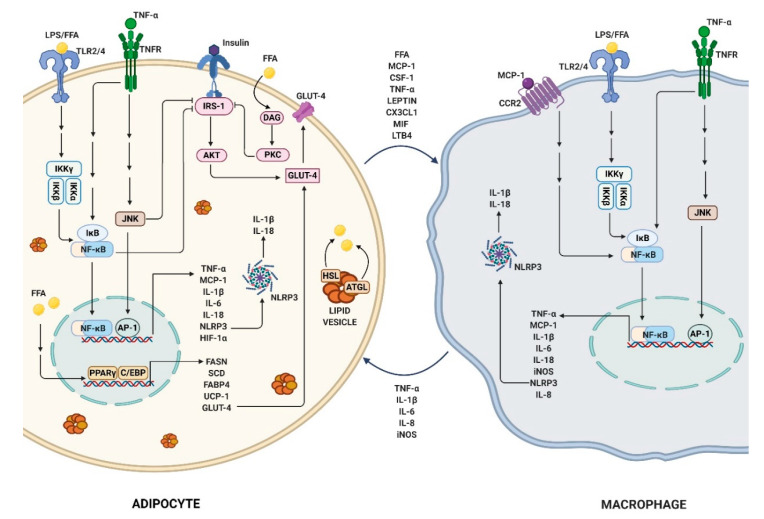
Adipocyte–macrophage crosstalk plays a key role in the induction and maintenance of obesity. Hypertrophic adipocytes release chemoattractants, promoting macrophage infiltration. Adipose-induced adipokines and free fatty acids (FFAs) stimulate adipose tissue macrophages (ATMs) into an M1 inflammatory stage to trigger JNK, NF-κB, and NLRP3-mediated pathways and inflammatory cytokines, which further induce adipocyte responses, including PPARγ and C/EBPs-regulated expressions of adipogenic, thermogenic, lipolytic, and lipogenic genes.

Several molecular pathways induce inflammation in obesity (Figure 2). TLR-mediated polarization of macrophages into an M1 inflammatory phenotype involves different transcription factors, such as NF-κB, PU.1, C/EBP-α, activator protein-1 (AP-1), STAT1, and interferon regulatory factor (IRF)-5 [82]. FFAs, TNF-α, or LPS can activate TLRs to stimulate c-Jun N-terminal kinases (JNK) or NF-κB mediated inflammation, resulting in enhanced innate immunity, activation of NLRP3 inflammasomes, and production of proinflammatory cytokines [83,84,85]. IKKβ and TLR4 deficiency in macrophages protected from insulin resistance in mice when exposed to HFD, and also inhibited FFA induced upregulation of TNF-α and IL-6 [54,86]. Mice, with a deficiency in TGF-β–activated kinase 1 (TAK1), an upstream modulator of NF-κB, in adipocytes, displayed an increased M2 ATM count in WAT, along with an enhanced resistance to HFD or leptin-deficiency-induced obesity [87]. Thus, targeting the IKKβ/NF-κB pathway has become an appealing approach to ameliorate the devastating effects of inflammation induced by obesity [88]. The association of obesity with increased insulin resistance has been extensively studied. Several adipokines such as leptin and retinol-binding protein (RBP)-4 were increased in obese insulin-resistant mice [33,89]. JNK and NF-κB pathways in adipocytes and macrophages, activated in response to obesity-induced stimuli, directly inhibit insulin response [63,90]. FFAs trigger diacylglycerol (DAG) and fatty acyl-CoA in the adipocytes, leading to protein kinase C (PKC) activation, which further phosphorylates insulin receptor substrate (IRS)-1 to inhibit AKT and GLUT-4, causing impairment in the ability of the liver to take up glucose and consequentially increased the circulating glucose levels (Figure 2) [91,92]. Obesity-induced inflammation and related dysregulated metabolic homeostasis often impact the liver, leading to nonalcoholic fatty liver diseases and steatosis (fat accumulation in the liver). Obesity enhances the supply of FFAs to the liver from the adipocytes, causing upregulation of PPARγ-dependent fatty acid translocase protein CD36. This increase in lipid storage elevates the activation of inflammatory responses in resident Kupffer cells and the recruitment of inflammatory myeloid cells to the liver, in a CCR2/MCP1-dependent manner, thereby elevating the severity of hepatic damage [93]. Evidence on the close overlap between the functional roles of adipocytes and macrophages imply inflammation to be the linking hub in obesity [94]. Therefore, defining, cell-specific regulators of obesity-induced inflammation can be promising in identifying therapeutic targets that can ameliorate the complications associated with obesity.

### 2.3. Obesity-Link Adipocyte and Macrophage Crosstalk

Adipocyte–macrophage crosstalk plays a central role in the induction and maintenance of obesity [95,96]. Macrophages constitute 40–60% of total cells in the adipose tissue depots of obese individuals, with an increase of ~5 fold, compared to lean [3,31]. Obese adipocyte-derived MCP-1, TNF-α and lipids, stimulate inflammatory monocyte infiltration and increase ATMs in adipose tissues [65,97]. Increased ATMs lead to higher levels of proinflammatory cytokines that cause further adipocyte dysregulation. ATM-derived TNF-α affects adipocytes by disrupting lipid and adipokine homeostasis, resulting in an increase of FFAs, among others. Adipocyte-induced FFAs further stimulate macrophages to express inflammatory cytokines. Hence, a disrupted macrophage–adipocyte crosstalk results in a harmful paracrine loop that exacerbates inflammation-mediated responses in the obese adipose tissue (Figure 2) [98,99,100]. Infiltrated ATMs in CLS are predominantly M1 phenotype, with just 10% in the outer rim of CLS accounting for M2 phenotype [39,43]. Initial studies suggested that adipocytes in CLS were highly necrotic in obese mice [101], while recent reports provide strong evidence that adipocytes undergo caspase-induced apoptosis [102,103]. Similar levels of adipocytes undergoing cell death were found in macrophage-depleted and control mice fed with HFD, suggesting that ATMs are not required for adipocyte cell death, rather the process is a response to elevated lipid dysregulation [104]. The ATMs in CLS function by taking up lipids, as well as removing the dead adipocytes through phagocytosis, and eventually form foam cells or become inactivated [105]. Further investigations on the understanding of the mechanistic nature of the macrophage–adipocytes crosstalk are much needed and guarantee to reveal vital knowledge, to help control obesity-induced inflammation.

## 3. Flavones and Their Impact on Obesity-Induced Inflammation

Obesity increases the incidence of heart disease by 30%, leads to diabetes, and is associated with cancer risk, as suggested by several meta-analyses [106]. Chronic inflammatory conditions represent one-third of the total $1.1 trillion US health care expenditure, representing approximately 20% of the annual national GDP. The currently used medications for obesity, such as orlistat, (a pancreatic lipase inhibitor), and liraglutide (an incretin mimetic), have severe side effects that accrue to other undesirable symptoms [107]. Hence, identifying additional approaches that lack unwanted effects is necessary.

Flavonoids are a large class of plant phenolic secondary metabolites with anti-obesogenic, anti-inflammatory, and immune-modulating activities. Higher consumption of flavonoid-rich diet has been linked to reduced energy consumption, food intake, and weight loss [108]. Thus, flavonoids might offer an economically favorable approach, with minimal, if any, side effects for the prevention and treatment of obesity. Flavonoids are structurally characterized by two benzene rings and a heterocyclic pyrone ring. Based on the oxidation and saturation status of the heterocyclic ring, flavonoids are categorized into different sub-groups, such as flavones, flavonols, flavanones, flavanonols, flavanols, isoflavones, and anthocyanidins [6]. Flavonoids are potent antioxidant agents and the molecular mechanisms by which they mitigate free-radical-derived oxidative stress have been extensively reported elsewhere [8,109]. The health beneficial effects of flavonoids are mediated primarily through their ability to modulate multiple gene/protein signaling networks. However, the basic mechanisms of action are not completely understood. Several studies support the beneficial role of flavonoids in obesity. The flavonol quercetin, perhaps one of the most studied in the context of obesity, increases adiponectin and downregulates MCP-1, TNF-α, and IL-6 expressions in adipocyte macrophage co-cultures and HFD mice models, via the inhibition of the NF-κB, AP-1, and mitogen-activated protein kinase (MAPK) pathways [110]. Resveratrol, a flavonoid found in red wine, decreased insulin resistance, inflammation, and CCR2-driven macrophage infiltration in SAT and VAT in HFD-fed mice [111]. Several studies have reported that soy isoflavones promote lipid homeostasis and fatty acid metabolism, and inhibit macrophage–adipocyte crosstalk both in vitro and in vivo [112,113]. Investigations on flavonoids as potential agents for treating obesity-linked cancers and obesity-associated modulation of gut microbiota are gaining interest. Resveratrol and naringenin suppressed inflammation and breast tumor growth by inhibiting adipocyte hypertrophy and tumor associated macrophages (TAM) in obese mice [114,115]. Consumption of anthocyanin containing foods can protect against diet-induced obesity and systemic inflammation, by modifying the gut microbial population in mice [116]. Striking associations of the dietary flavonoid intake with decreased obesity were found in numerous meta-analyses [117,118]. These findings established flavonoids as prospective arsenals in fighting obesity and reinforced the significance of their use in our daily diets and in clinical trials. Flavones, a sub-class of flavonoids, are highly efficacious as anti-inflammatory and anti-obesogenic agents. Here, we focus this review on the role of flavones in the prevention and treatment of obesity and its related disorders.

### 3.1. Flavone Sources and Structure

**Figure 3:**
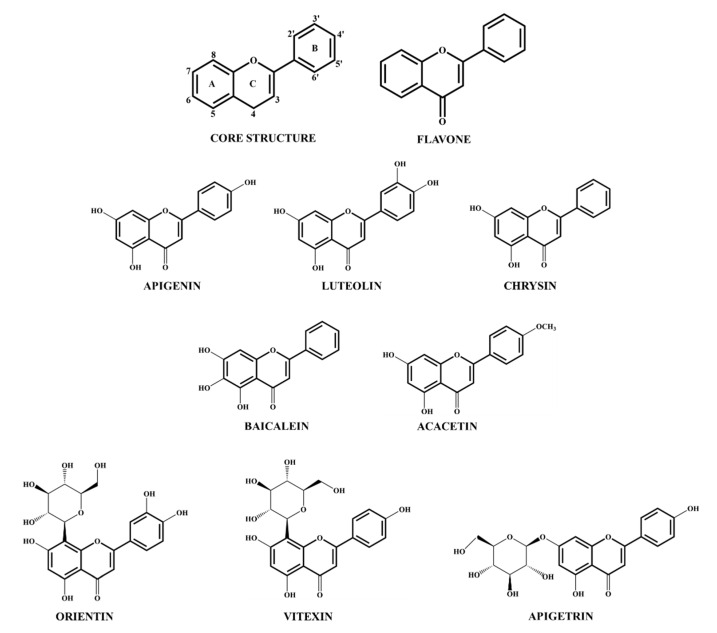
Structure of flavonoid core and different flavones.

Flavones are gaining immense interest due to their diverse bioactivity in plants and animals. They differ in structure from the other flavonoids in terms of the presence of a double-bond between C2 and C3 in the flavonoid core skeleton, a ketone at C4, and the absence of any modifications in the C3 position (Figure 3) [119]. The flavone core is subjected to substitutional conjugations, such as hydroxylations (addition of OH groups), glycosylations (bound to sugar moieties), or methoxylations (addition of methyl groups) at different positions, accounting for its expansive range of health beneficial activities [8]. Flavones are naturally found in plants as glucosides, conjugated either through hydroxyl groups (*O*-glycosides) or directly linked through the carbon (*C*-glycosides) groups. Functional activity, absorption, and bioavailability of these flavones can largely be dependent upon the structure, linkage, and the number of sugar moieties [120,121]. Most of the studies investigating the beneficial effects of flavones use them in their sugar-free form (aglycone). However, studies using whole foods with a high content of these phytochemical components in their naturally occurring form, remain scarce. We showed that aglycones are more easily absorbed than their glycosides, findings that are directly linked to their bioavailability and immunoregulatory functions [120]. Nevertheless, the poor solubility of aglycones imposes a great impediment for their clinical application in human health. We have overcome this gap in the field by developing foods from celery that increase the absorption and deliver bioactive concentrations of apigenin aglycone in vivo [120].

The levels of flavonoids can significantly vary between plants and tissues. The common food sources of flavones include citrus fruits, vegetables, herbs, and grains. Albeit flavones represent only a small fraction of the total flavonoid intake, it is estimated to range between 0.7 to 9.0 mg/day [122]. Rich, natural flavone sources are parsley, celery, peppermint, and sage, which predominantly contain apigenin and luteolin in their *O*-glucoside forms [123]. In maize, maysin and apimaysin are common flavones modified in –*C* groups. Another group with a wide array of physiological effects is the methoxylated flavones, such as acacetin, diosmetin, and chrysoeriol, which are commonly found in the citrus family [124]. Despite the available and procuring knowledge on the bioactivity of pure flavones, further investigations on the effect of whole foods containing a high flavone content need to be adopted, with rigorous consideration on the estimation of consumption quantity. This is vital for overcoming hurdles in accurately interpreting the association between flavone intake and health outcomes at clinical levels.

### 3.2. Role of Flavones in Obesity-Induced Inflammation

Several studies have suggested promising effects of flavones on the prevention and treatment of obesity-induced inflammation, based on their ability to modulate adipocyte, as well as their immune cell function. Flavones inhibit different stages of adipogenesis by suppressing lipid accumulation in adipocytes, through the reduction of lipogenesis and lipolysis (Table 1).

Apigenin and luteolin inhibit adipogenesis at 10–50 μM by attenuating the accumulation of intracellular triglycerides and the expression of adipogenic transcriptional factors, such as C/EBP and PPARγ, in differentiated 3T3-L1 and primary adipocytes, through the upregulation of 5′-adenosine monophosphate-activated protein kinase (AMPK) activity [125,133]. Apigenin and baicalein suppress proliferation and differentiation of preadipocytes, by inducing cell cycle arrest at G0/G1, and inhibiting MCE during the early stages of differentiation [126,137]. Lipid accumulation in mature human adipocytes and differentiated 3T3-L1 adipocytes was suppressed by apigenin, orientin (luteolin-8-*C*-glucoside), and baicalein. This suppression occurs through the reduction of lipolytic genes ATGL, HSL and monoacyl glyceride lipase (MGL), and lipogenic genes like fatty acid synthase (FASN), acetyl-CoA carboxylase (ACC) and stearoyl-CoA desaturase (SCD), which reflect on the anti-lipolytic and anti-lipogenic role of flavones [127,138,140]. However, apigenin had no impact on adipogenesis or the expression of any of adipogenic genes, including SREBP-1c in mature human mesenchymal stem cell-derived adipocytes, while chrysin enhanced lipolysis, adipogenesis, and lipogenesis in differentiated 3T3-L1 cells [127,141]. Differences in these findings could be attributed to the etiology, the stage of differentiation of the cells used and the treatment dose, thus, highlighting the need for additional studies and the use of models that fully capture the complex balance of physiological whole-body metabolism. Reduction in transcriptional and translational levels of other adipogenic genes, such as FABP4 and GLUT4, were observed in the differentiated 3T3-L1 adipocytes treated with orientin and baicalein [138,140]. Chrysin enhances WAT thermogenesis by upregulating the expression of UCP-1 [141]. Although flavone glycosides apigetrin (apigenin 7-*O*-glucoside) and vitexin (apigenin 8-*C*-glucoside) suppressed adipocyte differentiation in 3T3-L1 cells, through the activation of the ERK/MAPK pathway, the flavone concentrations used were significantly high and unreachable in vivo in mice or human clinical trials, thereby possibly masking the key underlying mechanisms [143,144].

Anti-obesity responses of flavones were further corroborated using HFD obese mice models (Table 1). Baicalin (20 mg/kg/day), luteolin (5 mg/kg/day), apigenin (50 mg/kg/day), and vitexin (5 mg/kg/day) repress the expression of transcription factors associated with adipose differentiation, attenuate adiposity, and mitigate lipogenesis in WAT of HFD-fed C57BL/6J mice [128,134,139,145]. Apigenin (15–30 mg/kg) inhibit preadipocyte differentiation and visceral obesity in HFD-fed mice, by directly interacting with STAT3, hence inhibiting STAT3 transcriptional activity and reducing the expression of CD36 and PPARγ [129]. These results further support that flavones can exert their biological activities through direct binding to proteins. We previously demonstrated that apigenin binds with different affinities to 160 proteins, by screening a human peptide phage display library coupled with next generation sequencing (PD-Seq) [149]. Among these targets, cathepsin D (CTSD), was implicated as a pivot mediator in adipogenesis, lipid metabolism in mouse hepatic steatosis, mitochondrial dysfunction, cell death, and macrophage infiltration, in hypertrophic adipose tissues of genetically and HFD-induced obese mouse models [150,151]. Future mechanistic studies are vital to reveal the mechanisms of action of flavones in obesity-induced inflammation to facilitate its prevention and therapeutics.

Flavones inhibit obesity-induced inflammation by reducing macrophage numbers in adipose tissues, thereby diminishing a proinflammatory adipose environment (Table 1). Apigenin and chrysin reduce the levels of proinflammatory cytokines IL-12, TNF-α, IL-6, and MCP-1 in adipose tissue in obese C57BL/6J mice [132,142]. This effect seems to be due to the ability of apigenin to switch macrophage phenotype from M1 to M2 by binding to PPARγ, thereby suppressing the interaction between PPARγ and NF-κB. Luteolin also decreases the infiltration of ATMs in the EAT of HFD-fed mice, by reversing the polarization of obesity-associated M1 and MMe ATMs through the activation of the AMPKα1 pathway [135]. Interesting studies using adipocyte-RAW 264.7 macrophages co-cultures and cell-specific conditioned media revealed that luteolin reduces inflammation by suppressing macrophage-stimulated inflammatory cytokines, but has no effect on adipocytes-stimulated adipokines, suggesting that luteolin specifically targets macrophages [152]. An alternative explanation of these results could probably owe to the use of hypertrophic adipocytes, as it was previously reported that only preadipocytes and adipocyte progenitors release chemokines such as MCP-1, to stimulate macrophage accumulation in adipose tissues, while their expression levels are low in mature adipocytes [153,154]. *Scutellaria baicalensis* roots rich in baicalin, wogonin, and luteolin alleviate HFD-induced insulin-resistance in obese mice, through modulation of inflammation, by promoting M2 phenotype skewing and reducing the TLR5 signaling pathway [136,146]. Flavones prevent non-alcoholic fatty liver disease (NAFLD) and steatosis. This effect seems to be due to the ability of flavones to increase liver fatty acid oxidation and reduce oxidative stress. Apigenin (50 mg/kg/day) reduces HFD/FFA-induced hepatic steatosis, lipid peroxidation, and lipid accumulation in the liver, by downregulating the lipogenic genes and inhibiting the overexpression of inflammatory markers and Kupffer macrophage infiltration [131]. These hepatic protective effects of apigenin were ascribed to its xanthine oxidase (a purine nucleotide degradation and ROS generator) inhibitor role, which hence inhibited the NLRP3 inflammasome assembly, ROS generation, and the release of inflammatory cytokines IL-1β and IL-18, illustrating a combined anti-oxidant and anti-inflammatory mechanism of action [131]. Oxidative stress was found to be mitigated by apigenin (30 mg/kg/day) and baicalin (50 mg/kg/day), by inhibiting the mitochondrial dysfunction through Nrf2 activation in adipocytes and macrophages in HFD/FFA-fed NAFLD mice [130,147]. Interestingly, induced activation of Nrf2 negatively regulated the PPARγ function in the NAFLD model, probably through direct interaction with Nrf2 [130]. Luteolin (5 mg/kg/day) decreases liver lipotoxicity by inducing FFAs flux to WAT and attenuates liver fibrosis by reducing cathepsin and extracellular matrix accumulation [136]. A quantitative proteomic study identified baicalin as an allosteric activator of carnitine palmitoyltransferase 1 (CPT1), the rate-limiting enzyme of fatty acid β-oxidation, wherein it significantly improved hepatic steatosis and decreased diet-induced obesity, by directly binding with CPT1 to facilitate accelerated lipid influx into the mitochondria for β-oxidation and FFA degradation [148]. These findings underscore the efficacious nature of flavones in tackling obesity-induced inflammation, by actively affecting both the inflammatory macrophages and the adipocytes in the adipose depots, and also their crosstalk. Despite a large number of encouraging studies suggesting the health beneficial impacts of flavones, it warrants further investigation of the different upstream molecular mechanisms of their roles in modulating obesity-induced inflammation, using foods rich in flavonoids, at feasible treatment doses.

### 3.3. Controlling Obesity-Associated Cancer Using Flavones

Obesity was positively correlated with cancer morbidity and mortality in both men and women [155]. In addition, preclinical and epidemiological studies implicated obesity as a major risk factor for the development of cancer [156,157,158]. This is especially significant in the case of breast cancer, where the adipose tissue is a predominant component of the stroma in the mammary tissue [159]. Cancer cells spread to stromal compartments that possess abundant adipose tissue, while adipocytes along with ATMs serve as a tumor-favoring niche with endocrine resources to nurture and mold the tumor microenvironment, contributing to tumor progression and metastasis [160,161]. The insulin–insulin growth factor (IGF)-1 axis, sex hormones, and adipokines are key mediators between obesity and cancer, each of which are tightly linked to the endocrine and paracrine dysregulation of adipose tissue in obese individuals [162]. For instance, adipose-derived stem cells secrete chemokine adipsin and promote breast cancer growth [163,164]. Leptin released from adipose tissue is shown to induce vascular endothelial growth factor (VEGF) overexpression and enhanced cancer stem cell-like properties in breast cancer [165,166]. VAT-derived fibroblast growth factor (FGF)-2 was reported to stimulate cell transformation through FGF receptor-1 in melanoma and breast cancer [167].

Hypertrophic expansion of adipose tissues in obese individuals shares many common aspects with tumor growth. Both obesity and cancer progression are closely associated with energy intake and nutrient availability. Hypoxia, often linked to obesity, stimulates enhanced angiogenesis, creating a microenvironment that provides a tumor permissive niche for the transformed or infiltrating cells [168,169]. Certain fibrotic factors such as adipose-derived collagen-IV and endotrophin are the key mediators linking obesity and tumor growth [170,171,172]. While adipocyte-released mediators are the predominant regulators of tumor progression, cancer cells can also induce metabolic differences and condition adipocytes in a pro-tumorigenic fashion, to form cancer-associated adipocytes (CAA) [173]. Paracrine signals from CAAs induce lipid degradation resulting in the release of FFAs, which is used as an energy source during metastasis by facilitating β-oxidation in cancer cells [174]. This reciprocal crosstalk between the cancer cells and adipocytes within the microenvironment is crucial for creating a tumor-permissive niche. Most importantly, both are characterized by chronic inflammation. Inflamed adipose tissues induce an aggravated expression of proinflammatory mediators, increased aromatase levels, and elevated estrogen receptor-α (ER-α)-dependent gene expression, which are also involved in tumor growth and metastasis [175,176]. Tumor cells also release MCP-1 to trigger macrophage infiltration, which are key contributions for tumor maintenance. While adipocytes recruit M1 phenotype macrophages, cancer cells skew macrophages towards an M2 phenotype [177]. Interestingly, adipocyte-cancer cell crosstalk was shown to influence chemotherapy efficacy and outcome in obese patients. The efficiency of tamoxifen to inhibit the proliferation of breast cancer cell line MCF-7 was significantly reduced in the presence of matured adipocytes derived from adipocyte stem cells of obese women. This effect was attributed to the increased presence of inflammatory adipokines, such as leptin, IL-6, and TNF-α, in the co-cultures of MCF-7 and adipocytes [178]. It is noteworthy that some of the key molecular players involved in obesity were also strikingly critical in cancer progression, such as NF-κB, CCL2/CCR2, JNK, and HIF/VEGF.

The anti-cancer efficacy of flavones relied on their ability to regulate key molecular pathways related to cancer cell proliferation and immune cell function, thereby halting tumor growth and metastasis [8]. Flavones inhibit cell growth and promote cell death in various cancer types. Importantly, we found that apigenin induces cell death of numerous cancer cell types but had no effect on the proliferation of non-cancer cells in leukemia [179]. We previously showed that apigenin induces cell cycle arrest by inducing DNA damage through the phosphorylation of ataxia-telangiectasia mutated kinase (ATM) and H2A histone family member X (H2AX) [180]. Luteolin induces apoptosis of colon cancer cells through its interaction with p53 and upregulation of Nrf2 [181]. In the human xenograft prostate cancer model, apigenin, through the inhibition of IGF/IGFR-1, reduced tumor growth [182]. Flavones can also suppress stem-like properties in aggressive cancers [183]. Baicalein inhibits the expression of stem cell markers CD44^high^CD24^low^ and octamer-binding transcription factors (OCT)-3 and 4 in triple negative breast cancer cell lines, through the inhibition of interferon-induced protein with tetratricopeptide repeats 2 (IFIT2) [184]. In addition, flavones are also potent immunoregulators. Apigenin and a celery-based apigenin-rich (CEBAR) food, a diet developed by our team that delivers in vivo effective doses of apigenin [120,185], reduce inflammation through the inhibition of NF-κB and decreased proinflammatory TNF-α in vivo [186,187]. Apigenin and luteolin suppress MCP-1 and IL-6 release, inhibiting TAM infiltration and migration of cancer cells [188,189,190]. These findings support the potent role of flavones in the prevention and treatment of obesity-induced cancers, and in enhancing the efficacy of chemotherapeutic drugs. However, further investigations on the effects of flavones on HFD-induced mammary tumorigenesis in preclinical PyMT mouse models are required, which can be significantly informative for clinical studies.

### 3.4. Flavones as Emerging Mediators of Gut Microbiota and Its Link with Obesity-Induced Inflammation

The gastrointestinal tract (GI) is inhabited by a broad repertoire of microorganisms, generically referred to as the gut microbiota. While the intestine provides a nutrient-rich, protected environment in which microbiota thrive to create a diverse and stable ecosystem, the microbiome provides nutrients to human host cells and prevent the entry of potential pathogens [191]. Microbes play an essential role in vitamin production, the modification of food components, energy homeostasis, intestinal mucosa formation, and the development of immunity. The gut microbiota interacts with the host cells through molecular communication, using small molecules and other metabolites [192]. Metagenomic analysis revealed that bacteria in the intestine belong to mainly three phyla, Bacteroidetes, Firmicutes, and Actinobacteria. Diet plays a drastic role in maintaining gut microbiota diversity [193]. An imbalance in the composition, richness, and the metabolic activity of gut microbiota, known as dysbiosis, can give rise to dramatic changes in the symbiotic relationship between the bacteria consortium and the host, leading to a variety of chronic disease conditions, including obesity [194]. Obese individuals have an altered gut microbiota diversity with a reduction in barrier-protecting microbes, such as Lactobacillus and Bifidobacterium, and promotion of opportunistic pathogenic bacterial abundances like the Enterobacteriaceae, Desulfovibrionaceae, and Streptococcaceae families [195,196]. Compelling evidence demonstrating the role of the gut microbiota in obesity was provided by germ-free (GF) mice fed with HFD, showing a lesser weight-gain than non-GF mice, observations that were a result of enhanced fatty acid metabolism in GF mice [197]. Dysbiosis was found in both HFD and genetically induced obese mice, as evidenced by a 50% increase in the *Firmicutes* species and a 50% decrease in the *Bacteroidetes* species in obese conditions [198,199]. Additionally, transplantation of gut microbiome from genetically obese donor mice into GF mice increased adiposity, as compared to GF mice that received gut microbes from lean mice [200]. Similarly, dysbiosis and a significant reduction in bacterial diversity characterized by a higher *Firmicutes* to *Bacteroidetes* ratio were also observed in obese humans [201]. In HFD-induced obesity, enhanced growth of Enterobacteriaceae was correlated with an increase in intestinal endotoxin production, conditions that are known to contribute to an inflammatory intestinal microenvironment [202]. Intestinal bacteria inhibit fasting-induced adipocyte factor, which affects lipase activity and enhance triglyceride deposition in adipocytes. Furthermore, the obesity-altered gut microbiota is potent at harvesting energy from food by secreting enzymes that break down nutrients more efficaciously [200]. Strong links between diet, inflammation, and microbial dysbiosis were found. Increased fat intake causes a rise in Gram-negative bacteria, augmenting circulatory LPS levels and weakening the intestinal gut endothelium junctions that lead to enhanced intestinal permeability. Higher levels of IFN-γ and IL-1β increased gut epithelial permeability by suppressing the expression of tight junction proteins like occludin [203]. The innate immune system plays a critical role in regulating the crosstalk between the host and the microbiota during obesity-induced inflammation. An increase of macrophage infiltration into the intestinal lamina propia was observed in obese conditions, resulting from similar molecular mechanisms responsible for ATM infiltration [204,205]. LPS-induced TLR and NLR mediated the JNK and NF-κB pathways in intestinal epithelial cells and macrophages and stimulated the production of proinflammatory cytokines, which further impaired intestinal permeability. An abundance of *Bacteroidetes* and *Akkermansia muciniphila* increased in TLR4 and NLRP6 transgenic mice, thereby altering the microbiota profile and reducing inflammation [206,207]. HFD induced intestinal NF-κB and TNF-α expression and enhanced adiposity, which was resisted by the GF mice. Interestingly, intestinal changes induced by the HFD and microbiota-derived inflammatory changes seem to precede the onset of obesity [208]. Changes in the gut microbiota composition of genetically obese mice was associated with decreased MCP-1 levels [209]. HFD-induced alterations in gut microbiota spectrum hampered gut barrier function and enhanced macrophage infiltration and inflammation in mesenteric fat, suggesting a link between microbiota and inflammation [5]. These studies confirm that the microbiota is the main hub controlling the inflammatory responses in the intestine. There is growing evidence that establishes the role of microbiota in stimulating obesity-induced cancers. Fecal transfer from HFD-fed mice with aggressive intestinal tumor to healthy K-ras^G12Dint^ mice led to a microbial community shift and enhanced tumor progression [210]. Transferring the microbiota from HFD-fed mice into female GF mice was associated with progressive hepatic cancer in the offspring [211]. Interestingly, *Akkermansia muciniphila* was identified to be associated with a favorable outcome in lung and renal cancer patients undergoing PD (programmed cell death protein)-1 immune checkpoint inhibitor chemotherapy, implicating the potential role of gut microbes in modulating host response to therapy [212].

The interplay between the gut microbiome and flavonoid metabolism is emerging as an important player in health [213,214]. The gut microbiota plays a key role in modulating the chemistry, bioavailability, and absorption of flavonoids. Intestinal microbial glycohydrolases, glucosidase, demethylation, dihydroxylation, and decarboxylation, modified flavonoids and the resulting metabolites were more efficiently absorbed in the intestine. This was evident from their increased enterohepatic and plasma levels, and the elevated biological functions, as compared to their precursors [215]. Glycoside forms of flavonoids are often converted to their aglycones when metabolized by the gut microbiota [216]. Quercetin produced from the microbiota-mediated transformation of quercitrin (quercetin-3-*O*-rhamnoside) exhibited higher anti-inflammatory responses through the inhibition of the NF-κB pathway [217]. On the other hand, flavonoids can induce changes in gut microbiome composition, after the consumption of foods with a high content of polyphenols, predominantly via inhibition of pathogenic microbes and stimulation of commensal microbes [218,219]. Flavonoids might stimulate commensal bacteria *Lactobacillus* and *Bifidobacterium* in the gut microbiota, while hampering the colonization of the pathogenic strain *Clostridium*, thereby, reducing the gut microbiota dysbiosis [220]. Therefore, the reciprocal mutual effects involving the transformation of flavonoids by the gut microbiota and the modulation of microbiota by flavonoid and its metabolites can profoundly impact the flavonoid bioavailability, biological effects, and ultimately human health.

So far, studies on the effect of flavones on gut microbiota are lacking. However, studies reporting the beneficial role of other flavonoids as potent gut microbiota modifiers are emerging. For example, quercetin reduced the microbiota composition including the *Firmicutes*/*Bacteroidetes* ratio and the growth of species associated with diet-induced obesity like *Erysipelotrichaceae*, *Bacillus*, and *Eubacterium cylindroides*, while restoring the barrier integrity in obese NAFLD model [221,222]. In HFD/high sucrose-induced obesity models, polyphenol-rich cranberry extract diet and concord grape anthocyanins inhibited insulin resistance and inflammation by mediating an increase in *Akkermansia muciniphila* in the gut microbiota [116,223]. Anthocyanins from plum and peach juices decreased fecal short-chain fatty acids (SCFA), a subset of key gut microbiota metabolites, including acetate, propionate, and butyrate, and modified the bacterial composition of the microbiota, by increasing the population of *Faecalibacterium*, *Lactobacillus*, and *Bacteroidetes* [224,225]. Several flavonoids are shown to enrich beneficial bacterial abundance while reducing potential detrimental microbes in the human gut. Quercetin and resveratrol decreased the Enterobacteriaceae family and reduced the *Firmicutes*/*Bacteroidetes* ratio in the human gut, as shown through the 16S rRNA sequencing of fecal samples [226,227]. Diet supplementation with soy bars significantly enhances the abundance of beneficial *Bifidobacterium* bacteria in postmenopausal women, which results in increased lipid catabolism [228]. The ability of other flavonoids to regulate dysbiosis establishes a promising platform, urging the investigation of the potential of flavones in the regulation of the gut microbiota.

Studies related to the effect of flavone apigenin on the gut microbiota are gaining interest. Apigenin suppressed colonic inflammation by reducing IL-1β and IL-6 and immune cell infiltration [9]. Using NLRP6^-/-^ mice and 16S rRNA gene sequencing of fecal samples, anti-inflammatory and anti-proliferative activity of apigenin was correlated to the apigenin-induced changes in the gut microbial composition, which was dependent on NLRP6 inflammasome. Notably, cohousing with apigenin-treated mice protected other mice against colitis, suggesting that the protective effects of apigenin were transmitted [229]. Apigenin inhibited *Enterococcus caccae* by upregulating genes pertaining to protein synthesis, DNA damage responses, and SCFA production, as identified by the 16S rRNA gene sequencing of apigenin-treated human fecal homogenates [230]. These findings suggest the potential role of apigenin as an active ingredient in modulating gut microbiota and hence mitigating obesity. To the best of our knowledge, studies pertaining to the effects of flavone intake on gut dysbiosis in humans remain to be reported. More studies considering the efficacy of various flavones in obese mouse models, as well as amongst humans within and between different regions, ethnicity, exercise regimes, and diets in regulating the intricate association between the gut microbiome and the immune system are necessary. Hence, an exhaustive understanding of the role of foods with a high content of flavones in the crosstalk between diet, gut microbiome, and immune system can provide a breakthrough in reducing obesity-induced inflammation.

## 4. Conclusions

Obesity and associated comorbidities have reached pandemic levels and require the identification of additional therapeutic and preventive approaches that lack adverse side effects and are cost-effective. Chronic inflammation has a crucial role in the initiation and maintenance of obesity, promoting metabolic dysregulation, microbiome dysbiosis, and increasing cancer incidence. A vicious crosstalk between adipocytes and the infiltrated immune cells led to a dramatic remodeling of the gene, protein, and lipid metabolic networks. Flavones, active plant metabolites or nutraceuticals, provide potential opportunities targeting numerous pathways that are central to obesity. The established evidence demonstrates that flavones ameliorate macrophage-mediated inflammation and reduce cancer progression and obesity. These recent findings underscore the efficacious nature of flavones in tackling obesity as a “sword of two edges”, targeting macrophages and adipocytes, thus, reestablishing homeostasis. Studies on the health beneficial impacts of flavones through the modulation of adipogenic and immunogenic regulators warrant further investigation and future exciting discoveries.

## Figures and Tables

**Table 1:** Flavones and their functional roles in obesity and its associated inflammation.

Flavone	Experimental Model	Concentration	Function	Reference
**Apigenin**	Mouse 3T3-L1 cells	10–50 μM	↓ adipogenesis: C/EBPβ and PPARγ ↓ lipolysis: HSL, MSL ↑ fatty acid oxidation: AMPK ↓ MCE, G0/G1 arrest	[125,126]
	Human mature adipocytes	25 μM	↓ lipogenesis: FASN No effect adipogenesis	[127]
	HFD-fed obese C57BL/6J mice	15–50 mg/kg/day	↓ adiposity ↓ lipogenesis: FASN ↑ lipolysis: ATGL, HSL ↑ fatty acid oxidation: AMPK and ACC ↓ inflammation: MAPK, NF-κB, TNF-α, IL-6 and MCP-1 ↓ ATM infiltration and M1 polarization ↑ thermogenesis: UCP-1 ↓ STAT3/CD36 ↓ liver steatosis and hepatic inflammation ↓ NLRP3 ↑ insulin sensitivity ↓ oxidative stress: XO and ROS ↑ Nrf2 activity	[128,129,130,131,132]
**Luteolin**	3T3-L1 cells	10–50 μM	↓ adipogenesis: C/EBPα and PPARγ ↓ lipogenesis	[133]
	HFD-fed obese C57BL/6J mice	5 mg/kg/day	↓ adiposity ↓ inflammation: IL-1β and IL-6 ↓ ATM infiltration and M1 polarization ↓ insulin resistance ↓ hepatic steatosis	[134,135,136]
**Baicalein**	3T3-L1 cells	12.5 μM	↓ adipogenesis: C/EBPα, C/EBPβ, FABP4 and PPARγ ↓ lipogenesis ↓ MCE, G0/G1 arrest	[137,138]
	Diet-induced obese C57BL/6J mice	20 mg/kg/day	↑ thermogenesis: UCP-1 ↑ insulin sensitivity: GLUT4	[139]
**Orientin**	3T3-L1 cells	50 μM	↓ adipogenesis: C/EBPα, C/EBPδ, PPARγ, FABP4 and GLUT4 ↓ lipogenesis: FASN, SCD, ACC ↓ lipolysis: HSL, MSL, ATGL ↓PI3K/Akt-FOXO1	[140]
**Chrysin**	3T3-L1 cells	50 μM	↑ adipogenesis: C/EBPα, C/EBPβ and PPARγ ↑ lipogenesis: ACC ↑ lipolysis: HSL, MSL, ↑ thermogenesis: UCP-1 ↑ AMPK	[141]
	Diet-induced obese C57BL/6J mice	20–30 mg/kg/day	↓ adiposity ↑ PPARγ ↓ inflammation: TNF-α, IL-6 and IL-1β ↓ ATM infiltration and M1 polarization	[142]
**Apigetrin**	3T3-L1 cells	100 μM	↓ adipogenesis: C/EBPα, PPARγ, and SREBP-1c ↓ lipogenesis: FASN ↓ inflammation: TNF-α and IL-6	[143]
**Vitexin**	3T3-L1 cells	25–100 μM	↓ adipogenesis: PPARγ ↓ lipogenesis ↑ ERK1/2 ↓ Akt	[144]
	HFD-fed obese C57BL/6 mice	5 mg/kg/day	↓ adiposity ↓ adipogenesis: C/EBPα and lipogenesis: FASN ↑ AMPK	[145]
**Wogonin** **(*Scutellaria baicalensis*)**	HFD-fed obese C57BL/6 mice	500 mg/kg/day	↓ insulin resistance ↓ inflammation: TNF-α and IFN-γ	[146]
**Baicalin**	HFD-fed obese C57BL/6 mice	5 mg/kg/day	↑ insulin sensitivity ↓ inflammation: TNF-α, MCP-1 and IL-1β ↓ oxidative stress ↑ Nrf2 activity ↑ CPT1A activity	[147,148]

## References

[1] Bluher M. (2019). Obesity: Global epidemiology and pathogenesis. Nat. Rev. Endocrinol..

[2] Reilly S.M., Saltiel A.R. (2017). Adapting to obesity with adipose tissue inflammation. Nat. Rev. Endocrinol..

[3] Weisberg S.P., McCann D., Desai M., Rosenbaum M., Leibel R.L., Ferrante A.W. (2003). Obesity is associated with macrophage accumulation in adipose tissue. J. Clin. Investig..

[4] Liu R., Nikolajczyk B.S. (2019). Tissue Immune Cells Fuel Obesity-Associated Inflammation in Adipose Tissue and Beyond. Front. Immunol..

[5] Lam Y.Y., Ha C.W., Campbell C.R., Mitchell A.J., Dinudom A., Oscarsson J., Cook D.I., Hunt N.H., Caterson I.D., Holmes A.J. (2012). Increased gut permeability and microbiota change associate with mesenteric fat inflammation and metabolic dysfunction in diet-induced obese mice. PLoS ONE.

[6] Jiang N., Doseff A.I., Grotewold E. (2016). Flavones: From Biosynthesis to Health Benefits. Plants.

[7] Panche A.N., Diwan A.D., Chandra S.R. (2016). Flavonoids: An overview. J. Nutr. Sci..

[8] Sudhakaran M., Sardesai S., Doseff A.I. (2019). Flavonoids: New Frontier for Immuno-Regulation and Breast Cancer Control. Antioxidants.

[9] Gentile D., Fornai M., Colucci R., Pellegrini C., Tirotta E., Benvenuti L., Segnani C., Ippolito C., Duranti E., Virdis A. (2018). The flavonoid compound apigenin prevents colonic inflammation and motor dysfunctions associated with high fat diet-induced obesity. PLoS ONE.

[10] Vernarelli J.A., Lambert J.D. (2017). Flavonoid intake is inversely associated with obesity and C-reactive protein, a marker for inflammation, in US adults. Nutr. Diabetes.

[11] Gil-Cardoso K., Gines I., Pinent M., Ardevol A., Blay M., Terra X. (2016). Effects of flavonoids on intestinal inflammation, barrier integrity and changes in gut microbiota during diet-induced obesity. Nutr. Res. Rev..

[12] Choe S.S., Huh J.Y., Hwang I.J., Kim J.I., Kim J.B. (2016). Adipose Tissue Remodeling: Its Role in Energy Metabolism and Metabolic Disorders. Front. Endocrinol. (Lausanne).

[13] Shinoda K., Luijten I.H., Hasegawa Y., Hong H., Sonne S.B., Kim M., Xue R., Chondronikola M., Cypess A.M., Tseng Y.H. (2015). Genetic and functional characterization of clonally derived adult human brown adipocytes. Nat. Med..

[14] Schweiger M., Schreiber R., Haemmerle G., Lass A., Fledelius C., Jacobsen P., Tornqvist H., Zechner R., Zimmermann R. (2006). Adipose triglyceride lipase and hormone-sensitive lipase are the major enzymes in adipose tissue triacylglycerol catabolism. J. Biol. Chem..

[15] Scherer P.E. (2006). Adipose tissue: From lipid storage compartment to endocrine organ. Diabetes.

[16] Cao H., Gerhold K., Mayers J.R., Wiest M.M., Watkins S.M., Hotamisligil G.S. (2008). Identification of a lipokine, a lipid hormone linking adipose tissue to systemic metabolism. Cell.

[17] Scarpace P.J., Zhang Y. (2009). Leptin resistance: A prediposing factor for diet-induced obesity. Am. J. Physiol. Regul. Integr. Comp. Physiol..

[18] Yamauchi T., Kamon J., Minokoshi Y., Ito Y., Waki H., Uchida S., Yamashita S., Noda M., Kita S., Ueki K. (2002). Adiponectin stimulates glucose utilization and fatty-acid oxidation by activating AMP-activated protein kinase. Nat. Med..

[19] Min S.Y., Desai A., Yang Z., Sharma A., DeSouza T., Genga R.M.J., Kucukural A., Lifshitz L.M., Nielsen S., Scheele C. (2019). Diverse repertoire of human adipocyte subtypes develops from transcriptionally distinct mesenchymal progenitor cells. Proc. Natl. Acad. Sci. USA.

[20] Raajendiran A., Ooi G., Bayliss J., O′Brien P.E., Schittenhelm R.B., Clark A.K., Taylor R.A., Rodeheffer M.S., Burton P.R., Watt M.J. (2019). Identification of Metabolically Distinct Adipocyte Progenitor Cells in Human Adipose Tissues. Cell Rep..

[21] Rosen E.D., MacDougald O.A. (2006). Adipocyte differentiation from the inside out. Nat. Rev. Mol. Cell Biol..

[22] Otto T.C., Lane M.D. (2005). Adipose development: From stem cell to adipocyte. Crit. Rev. Biochem. Mol. Biol..

[23] Farmer S.R. (2006). Transcriptional control of adipocyte formation. Cell Metab..

[24] Rosen E.D., Walkey C.J., Puigserver P., Spiegelman B.M. (2000). Transcriptional regulation of adipogenesis. Genes Dev..

[25] Tontonoz P., Hu E., Spiegelman B.M. (1994). Stimulation of adipogenesis in fibroblasts by PPAR gamma 2, a lipid-activated transcription factor. Cell.

[26] Ehrlund A., Mejhert N., Bjork C., Andersson R., Kulyte A., Astrom G., Itoh M., Kawaji H., Lassmann T., Daub C.O. (2017). Transcriptional Dynamics during Human Adipogenesis and Its Link to Adipose Morphology and Distribution. Diabetes.

[27] Ahn J., Wu H., Lee K. (2019). Integrative Analysis Revealing Human Adipose-Specific Genes and Consolidating Obesity Loci. Sci. Rep..

[28] Sun K., Kusminski C.M., Scherer P.E. (2011). Adipose tissue remodeling and obesity. J. Clin. Investig..

[29] Kang K., Reilly S.M., Karabacak V., Gangl M.R., Fitzgerald K., Hatano B., Lee C.H. (2008). Adipocyte-derived Th2 cytokines and myeloid PPARdelta regulate macrophage polarization and insulin sensitivity. Cell Metab..

[30] Fujisaka S., Usui I., Bukhari A., Ikutani M., Oya T., Kanatani Y., Tsuneyama K., Nagai Y., Takatsu K., Urakaze M. (2009). Regulatory mechanisms for adipose tissue M1 and M2 macrophages in diet-induced obese mice. Diabetes.

[31] Oh D.Y., Morinaga H., Talukdar S., Bae E.J., Olefsky J.M. (2012). Increased macrophage migration into adipose tissue in obese mice. Diabetes.

[32] Lumeng C.N., Deyoung S.M., Bodzin J.L., Saltiel A.R. (2007). Increased inflammatory properties of adipose tissue macrophages recruited during diet-induced obesity. Diabetes.

[33] Xu H., Barnes G.T., Yang Q., Tan G., Yang D., Chou C.J., Sole J., Nichols A., Ross J.S., Tartaglia L.A. (2003). Chronic inflammation in fat plays a crucial role in the development of obesity-related insulin resistance. J. Clin. Investig..

[34] Kanda H., Tateya S., Tamori Y., Kotani K., Hiasa K., Kitazawa R., Kitazawa S., Miyachi H., Maeda S., Egashira K. (2006). MCP-1 contributes to macrophage infiltration into adipose tissue, insulin resistance, and hepatic steatosis in obesity. J. Clin. Investig..

[35] Zheng C., Yang Q., Xu C., Shou P., Cao J., Jiang M., Chen Q., Cao G., Han Y., Li F. (2015). CD11b regulates obesity-induced insulin resistance via limiting alternative activation and proliferation of adipose tissue macrophages. Proc. Natl. Acad. Sci. USA.

[36] Shah R., Hinkle C.C., Ferguson J.F., Mehta N.N., Li M., Qu L., Lu Y., Putt M.E., Ahima R.S., Reilly M.P. (2011). Fractalkine is a novel human adipochemokine associated with type 2 diabetes. Diabetes.

[37] Spite M., Hellmann J., Tang Y., Mathis S.P., Kosuri M., Bhatnagar A., Jala V.R., Haribabu B. (2011). Deficiency of the leukotriene B4 receptor, BLT-1, protects against systemic insulin resistance in diet-induced obesity. J. Immunol..

[38] Finucane O.M., Reynolds C.M., McGillicuddy F.C., Harford K.A., Morrison M., Baugh J., Roche H.M. (2014). Macrophage migration inhibitory factor deficiency ameliorates high-fat diet induced insulin resistance in mice with reduced adipose inflammation and hepatic steatosis. PLoS ONE.

[39] Haase J., Weyer U., Immig K., Kloting N., Bluher M., Eilers J., Bechmann I., Gericke M. (2014). Local proliferation of macrophages in adipose tissue during obesity-induced inflammation. Diabetologia.

[40] Zheng C., Yang Q., Cao J., Xie N., Liu K., Shou P., Qian F., Wang Y., Shi Y. (2016). Local proliferation initiates macrophage accumulation in adipose tissue during obesity. Cell Death Dis..

[41] Kratz M., Coats B.R., Hisert K.B., Hagman D., Mutskov V., Peris E., Schoenfelt K.Q., Kuzma J.N., Larson I., Billing P.S. (2014). Metabolic dysfunction drives a mechanistically distinct proinflammatory phenotype in adipose tissue macrophages. Cell Metab..

[42] Xu X., Grijalva A., Skowronski A., van Eijk M., Serlie M.J., Ferrante A.W. (2013). Obesity activates a program of lysosomal-dependent lipid metabolism in adipose tissue macrophages independently of classic activation. Cell Metab..

[43] Lumeng C.N., DelProposto J.B., Westcott D.J., Saltiel A.R. (2008). Phenotypic switching of adipose tissue macrophages with obesity is generated by spatiotemporal differences in macrophage subtypes. Diabetes.

[44] Feuerer M., Herrero L., Cipolletta D., Naaz A., Wong J., Nayer A., Lee J., Goldfine A.B., Benoist C., Shoelson S. (2009). Lean, but not obese, fat is enriched for a unique population of regulatory T cells that affect metabolic parameters. Nat. Med..

[45] Liu G., Ma H., Qiu L., Li L., Cao Y., Ma J., Zhao Y. (2011). Phenotypic and functional switch of macrophages induced by regulatory CD4+CD25+ T cells in mice. Immunol. Cell Biol..

[46] Qiu Y., Nguyen K.D., Odegaard J.I., Cui X., Tian X., Locksley R.M., Palmiter R.D., Chawla A. (2014). Eosinophils and type 2 cytokine signaling in macrophages orchestrate development of functional beige fat. Cell.

[47] Brestoff J.R., Kim B.S., Saenz S.A., Stine R.R., Monticelli L.A., Sonnenberg G.F., Thome J.J., Farber D.L., Lutfy K., Seale P. (2015). Group 2 innate lymphoid cells promote beiging of white adipose tissue and limit obesity. Nature.

[48] Chen H.H., Tseng Y.J., Wang S.Y., Tsai Y.S., Chang C.S., Kuo T.C., Yao W.J., Shieh C.C., Wu C.H., Kuo P.H. (2015). The metabolome profiling and pathway analysis in metabolic healthy and abnormal obesity. Int. J. Obes. (Lond.).

[49] Eisinger K., Krautbauer S., Hebel T., Schmitz G., Aslanidis C., Liebisch G., Buechler C. (2014). Lipidomic analysis of the liver from high-fat diet induced obese mice identifies changes in multiple lipid classes. Exp. Mol. Pathol..

[50] Hayakawa J., Wang M., Wang C., Han R.H., Jiang Z.Y., Han X. (2018). Lipidomic analysis reveals significant lipogenesis and accumulation of lipotoxic components in ob/ob mouse organs. Prostaglandins Leukot. Essent. Fat. Acids.

[51] Wang H., Ye J. (2015). Regulation of energy balance by inflammation: Common theme in physiology and pathology. Rev. Endocr. Metab. Disord..

[52] Cani P.D., Amar J., Iglesias M.A., Poggi M., Knauf C., Bastelica D., Neyrinck A.M., Fava F., Tuohy K.M., Chabo C. (2007). Metabolic endotoxemia initiates obesity and insulin resistance. Diabetes.

[53] Cani P.D., Jordan B.F. (2018). Gut microbiota-mediated inflammation in obesity: A link with gastrointestinal cancer. Nat. Rev. Gastroenterol. Hepatol..

[54] Shi H., Kokoeva M.V., Inouye K., Tzameli I., Yin H., Flier J.S. (2006). TLR4 links innate immunity and fatty acid-induced insulin resistance. J. Clin. Investig..

[55] Jernas M., Palming J., Sjoholm K., Jennische E., Svensson P.A., Gabrielsson B.G., Levin M., Sjogren A., Rudemo M., Lystig T.C. (2006). Separation of human adipocytes by size: Hypertrophic fat cells display distinct gene expression. FASEB J..

[56] Lee Y.S., Kim J.W., Osborne O., Oh D.Y., Sasik R., Schenk S., Chen A., Chung H., Murphy A., Watkins S.M. (2014). Increased adipocyte O2 consumption triggers HIF-1alpha, causing inflammation and insulin resistance in obesity. Cell.

[57] Strissel K.J., Stancheva Z., Miyoshi H., Perfield J.W., DeFuria J., Jick Z., Greenberg A.S., Obin M.S. (2007). Adipocyte death, adipose tissue remodeling, and obesity complications. Diabetes.

[58] Vandanmagsar B., Youm Y.H., Ravussin A., Galgani J.E., Stadler K., Mynatt R.L., Ravussin E., Stephens J.M., Dixit V.D. (2011). The NLRP3 inflammasome instigates obesity-induced inflammation and insulin resistance. Nat. Med..

[59] Furukawa S., Fujita T., Shimabukuro M., Iwaki M., Yamada Y., Nakajima Y., Nakayama O., Makishima M., Matsuda M., Shimomura I. (2004). Increased oxidative stress in obesity and its impact on metabolic syndrome. J. Clin. Investig..

[60] Amano S.U., Cohen J.L., Vangala P., Tencerova M., Nicoloro S.M., Yawe J.C., Shen Y., Czech M.P., Aouadi M. (2014). Local proliferation of macrophages contributes to obesity-associated adipose tissue inflammation. Cell Metab..

[61] Feng B., Jiao P., Nie Y., Kim T., Jun D., van Rooijen N., Yang Z., Xu H. (2011). Clodronate liposomes improve metabolic profile and reduce visceral adipose macrophage content in diet-induced obese mice. PLoS ONE.

[62] Kim D.H., Sandoval D., Reed J.A., Matter E.K., Tolod E.G., Woods S.C., Seeley R.J. (2008). The role of GM-CSF in adipose tissue inflammation. Am. J. Physiol. Endocrinol. Metab..

[63] Ramkhelawon B., Hennessy E.J., Menager M., Ray T.D., Sheedy F.J., Hutchison S., Wanschel A., Oldebeken S., Geoffrion M., Spiro W. (2014). Netrin-1 promotes adipose tissue macrophage retention and insulin resistance in obesity. Nat. Med..

[64] McLaughlin T., Ackerman S.E., Shen L., Engleman E. (2017). Role of innate and adaptive immunity in obesity-associated metabolic disease. J. Clin. Investig..

[65] Lee C.H., Lam K.S. (2019). Obesity-induced insulin resistance and macrophage infiltration of the adipose tissue: A vicious cycle. J. Diabetes Investig..

[66] Murano I., Barbatelli G., Parisani V., Latini C., Muzzonigro G., Castellucci M., Cinti S. (2008). Dead adipocytes, detected as crown-like structures, are prevalent in visceral fat depots of genetically obese mice. J. Lipid Res..

[67] McLaughlin T., Liu L.F., Lamendola C., Shen L., Morton J., Rivas H., Winer D., Tolentino L., Choi O., Zhang H. (2014). T-cell profile in adipose tissue is associated with insulin resistance and systemic inflammation in humans. Arterioscler. Thromb. Vasc. Biol..

[68] Nishimura S., Manabe I., Nagasaki M., Eto K., Yamashita H., Ohsugi M., Otsu M., Hara K., Ueki K., Sugiura S. (2009). CD8+ effector T cells contribute to macrophage recruitment and adipose tissue inflammation in obesity. Nat. Med..

[69] Deng T., Lyon C.J., Minze L.J., Lin J., Zou J., Liu J.Z., Ren Y., Yin Z., Hamilton D.J., Reardon P.R. (2013). Class II major histocompatibility complex plays an essential role in obesity-induced adipose inflammation. Cell Metab..

[70] Zou J., Lai B., Zheng M., Chen Q., Jiang S., Song A., Huang Z., Shi P., Tu X., Wang D. (2018). CD4+ T cells memorize obesity and promote weight regain. Cell. Mol. Immunol..

[71] DeFuria J., Belkina A.C., Jagannathan-Bogdan M., Snyder-Cappione J., Carr J.D., Nersesova Y.R., Markham D., Strissel K.J., Watkins A.A., Zhu M. (2013). B cells promote inflammation in obesity and type 2 diabetes through regulation of T-cell function and an inflammatory cytokine profile. Proc. Natl. Acad. Sci. USA.

[72] Duffaut C., Galitzky J., Lafontan M., Bouloumie A. (2009). Unexpected trafficking of immune cells within the adipose tissue during the onset of obesity. Biochem. Biophys. Res. Commun..

[73] Rajbhandari P., Arneson D., Hart S.K., Ahn I.S., Diamante G., Santos L.C., Zaghari N., Feng A.C., Thomas B.J., Vergnes L. (2019). Single cell analysis reveals immune cell-adipocyte crosstalk regulating the transcription of thermogenic adipocytes. Elife.

[74] Zhang X., Wang X., Yin H., Zhang L., Feng A., Zhang Q.X., Lin Y., Bao B., Hernandez L.L., Shi G.P. (2019). Functional Inactivation of Mast Cells Enhances Subcutaneous Adipose Tissue Browning in Mice. Cell Rep..

[75] Lee E.H., Itan M., Jang J., Gu H.J., Rozenberg P., Mingler M.K., Wen T., Yoon J., Park S.Y., Roh J.Y. (2018). Eosinophils support adipocyte maturation and promote glucose tolerance in obesity. Sci. Rep..

[76] Wu D., Molofsky A.B., Liang H.E., Ricardo-Gonzalez R.R., Jouihan H.A., Bando J.K., Chawla A., Locksley R.M. (2011). Eosinophils sustain adipose alternatively activated macrophages associated with glucose homeostasis. Science.

[77] Talukdar S., Oh D.Y., Bandyopadhyay G., Li D., Xu J., McNelis J., Lu M., Li P., Yan Q., Zhu Y. (2012). Neutrophils mediate insulin resistance in mice fed a high-fat diet through secreted elastase. Nat. Med..

[78] Bertola A., Ciucci T., Rousseau D., Bourlier V., Duffaut C., Bonnafous S., Blin-Wakkach C., Anty R., Iannelli A., Gugenheim J. (2012). Identification of adipose tissue dendritic cells correlated with obesity-associated insulin-resistance and inducing Th17 responses in mice and patients. Diabetes.

[79] Cho K.W., Zamarron B.F., Muir L.A., Singer K., Porsche C.E., DelProposto J.B., Geletka L., Meyer K.A., O’Rourke R.W., Lumeng C.N. (2016). Adipose Tissue Dendritic Cells Are Independent Contributors to Obesity-Induced Inflammation and Insulin Resistance. J. Immunol..

[80] Molofsky A.B., Nussbaum J.C., Liang H.E., Van Dyken S.J., Cheng L.E., Mohapatra A., Chawla A., Locksley R.M. (2013). Innate lymphoid type 2 cells sustain visceral adipose tissue eosinophils and alternatively activated macrophages. J. Exp. Med..

[81] Sasaki T., Moro K., Kubota T., Kubota N., Kato T., Ohno H., Nakae S., Saito H., Koyasu S. (2019). Innate Lymphoid Cells in the Induction of Obesity. Cell Rep..

[82] Juhas U., Ryba-Stanislawowska M., Szargiej P., Mysliwska J. (2015). Different pathways of macrophage activation and polarization. Postępy Hig. Med. Dośw. (Online).

[83] Suganami T., Tanimoto-Koyama K., Nishida J., Itoh M., Yuan X., Mizuarai S., Kotani H., Yamaoka S., Miyake K., Aoe S. (2007). Role of the Toll-like receptor 4/NF-kappaB pathway in saturated fatty acid-induced inflammatory changes in the interaction between adipocytes and macrophages. Arterioscler. Thromb. Vasc. Biol..

[84] Pal D., Dasgupta S., Kundu R., Maitra S., Das G., Mukhopadhyay S., Ray S., Majumdar S., Bhattacharya S. (2012). Fetuin-A acts as an endogenous ligand of TLR4 to promote lipid-induced insulin resistance. Nat. Med..

[85] Wen H., Gris D., Lei Y., Jha S., Zhang L., Huang M.T., Brickey W.J., Ting J.P. (2011). Fatty acid-induced NLRP3-ASC inflammasome activation interferes with insulin signaling. Nat. Immunol..

[86] Arkan M.C., Hevener A.L., Greten F.R., Maeda S., Li Z.W., Long J.M., Wynshaw-Boris A., Poli G., Olefsky J., Karin M. (2005). IKK-beta links inflammation to obesity-induced insulin resistance. Nat. Med..

[87] Sassmann-Schweda A., Singh P., Tang C., Wietelmann A., Wettschureck N., Offermanns S. (2016). Increased apoptosis and browning of TAK1-deficient adipocytes protects against obesity. JCI Insight.

[88] Gaestel M., Kotlyarov A., Kracht M. (2009). Targeting innate immunity protein kinase signalling in inflammation. Nat. Rev. Drug Discov..

[89] Yang Q., Graham T.E., Mody N., Preitner F., Peroni O.D., Zabolotny J.M., Kotani K., Quadro L., Kahn B.B. (2005). Serum retinol binding protein 4 contributes to insulin resistance in obesity and type 2 diabetes. Nature.

[90] Odegaard J.I., Ricardo-Gonzalez R.R., Goforth M.H., Morel C.R., Subramanian V., Mukundan L., Red Eagle A., Vats D., Brombacher F., Ferrante A.W. (2007). Macrophage-specific PPARgamma controls alternative activation and improves insulin resistance. Nature.

[91] Aguirre V., Uchida T., Yenush L., Davis R., White M.F. (2000). The c-Jun NH(2)-terminal kinase promotes insulin resistance during association with insulin receptor substrate-1 and phosphorylation of Ser(307). J. Biol. Chem..

[92] Gao Z., Hwang D., Bataille F., Lefevre M., York D., Quon M.J., Ye J. (2002). Serine phosphorylation of insulin receptor substrate 1 by inhibitor kappa B kinase complex. J. Biol. Chem..

[93] Obstfeld A.E., Sugaru E., Thearle M., Francisco A.M., Gayet C., Ginsberg H.N., Ables E.V., Ferrante A.W. (2010). C-C chemokine receptor 2 (CCR2) regulates the hepatic recruitment of myeloid cells that promote obesity-induced hepatic steatosis. Diabetes.

[94] Charriere G., Cousin B., Arnaud E., Andre M., Bacou F., Penicaud L., Casteilla L. (2003). Preadipocyte conversion to macrophage. Evidence of plasticity. J. Biol. Chem..

[95] Russo L., Lumeng C.N. (2018). Properties and functions of adipose tissue macrophages in obesity. Immunology.

[96] Huh J.Y., Park Y.J., Ham M., Kim J.B. (2014). Crosstalk between adipocytes and immune cells in adipose tissue inflammation and metabolic dysregulation in obesity. Mol. Cells.

[97] Kamei N., Tobe K., Suzuki R., Ohsugi M., Watanabe T., Kubota N., Ohtsuka-Kowatari N., Kumagai K., Sakamoto K., Kobayashi M. (2006). Overexpression of monocyte chemoattractant protein-1 in adipose tissues causes macrophage recruitment and insulin resistance. J. Biol. Chem..

[98] Wellen K.E., Hotamisligil G.S. (2003). Obesity-induced inflammatory changes in adipose tissue. J. Clin. Investig..

[99] Suganami T., Nishida J., Ogawa Y. (2005). A paracrine loop between adipocytes and macrophages aggravates inflammatory changes: Role of free fatty acids and tumor necrosis factor α. Arterioscler. Thromb. Vasc. Biol..

[100] Saltiel A.R., Olefsky J.M. (2017). Inflammatory mechanisms linking obesity and metabolic disease. J. Clin. Investig..

[101] Cinti S., Mitchell G., Barbatelli G., Murano I., Ceresi E., Faloia E., Wang S., Fortier M., Greenberg A.S., Obin M.S. (2005). Adipocyte death defines macrophage localization and function in adipose tissue of obese mice and humans. J. Lipid Res..

[102] Alkhouri N., Gornicka A., Berk M.P., Thapaliya S., Dixon L.J., Kashyap S., Schauer P.R., Feldstein A.E. (2010). Adipocyte apoptosis, a link between obesity, insulin resistance, and hepatic steatosis. J. Biol. Chem..

[103] Keuper M., Bluher M., Schon M.R., Moller P., Dzyakanchuk A., Amrein K., Debatin K.M., Wabitsch M., Fischer-Posovszky P. (2011). An inflammatory micro-environment promotes human adipocyte apoptosis. Mol. Cell. Endocrinol..

[104] Feng D., Tang Y., Kwon H., Zong H., Hawkins M., Kitsis R.N., Pessin J.E. (2011). High-fat diet-induced adipocyte cell death occurs through a cyclophilin D intrinsic signaling pathway independent of adipose tissue inflammation. Diabetes.

[105] Shapiro H., Pecht T., Shaco-Levy R., Harman-Boehm I., Kirshtein B., Kuperman Y., Chen A., Bluher M., Shai I., Rudich A. (2013). Adipose tissue foam cells are present in human obesity. J. Clin. Endocrinol. Metab..

[106] Bogers R.P., Bemelmans W.J.E., Hoogenveen R.T., Boshuizen H.C., Woodward M., Knekt P., van Dam R.M., Hu F.B., Visscher T.L.S., Menotti A. (2007). Association of Overweight with Increased Risk of Coronary Heart Disease Partly Independent of Blood Pressure and Cholesterol Levels: A Meta-analysis of 21 Cohort Studies Including More Than 300,000 Persons. Arch. Intern. Med..

[107] Li M.F., Cheung B.M. (2011). Rise and fall of anti-obesity drugs. World J. Diabetes.

[108] Bertoia M.L., Rimm E.B., Mukamal K.J., Hu F.B., Willett W.C., Cassidy A. (2016). Dietary flavonoid intake and weight maintenance: three prospective cohorts of 124,086 US men and women followed for up to 24 years. BMJ.

[109] Pietta P.-G. (2000). Flavonoids as Antioxidants. J. Nat. Prod..

[110] Seo M.J., Lee Y.J., Hwang J.H., Kim K.J., Lee B.Y. (2015). The inhibitory effects of quercetin on obesity and obesity-induced inflammation by regulation of MAPK signaling. J. Nutr. Biochem..

[111] Ding S., Jiang J., Wang Z., Zhang G., Yin J., Wang X., Wang S., Yu Z. (2018). Resveratrol reduces the inflammatory response in adipose tissue and improves adipose insulin signaling in high-fat diet-fed mice. PeerJ.

[112] Sakamoto Y., Kanatsu J., Toh M., Naka A., Kondo K., Iida K. (2016). The Dietary Isoflavone Daidzein Reduces Expression of Pro-Inflammatory Genes through PPARalpha/gamma and JNK Pathways in Adipocyte and Macrophage Co-Cultures. PLoS ONE.

[113] Tan J., Huang C., Luo Q., Liu W., Cheng D., Li Y., Xia Y., Li C., Tang L., Fang J. (2019). Soy Isoflavones Ameliorate Fatty Acid Metabolism of Visceral Adipose Tissue by Increasing the AMPK Activity in Male Rats with Diet-Induced Obesity (DIO). Molecules.

[114] Ke J.Y., Banh T., Hsiao Y.H., Cole R.M., Straka S.R., Yee L.D., Belury M.A. (2017). Citrus flavonoid naringenin reduces mammary tumor cell viability, adipose mass, and adipose inflammation in obese ovariectomized mice. Mol. Nutr. Food Res..

[115] Rossi E.L., Khatib S.A., Doerstling S.S., Bowers L.W., Pruski M., Ford N.A., Glickman R.D., Niu M., Yang P., Cui Z. (2018). Resveratrol inhibits obesity-associated adipose tissue dysfunction and tumor growth in a mouse model of postmenopausal claudin-low breast cancer. Mol. Carcinog..

[116] Roopchand D.E., Carmody R.N., Kuhn P., Moskal K., Rojas-Silva P., Turnbaugh P.J., Raskin I. (2015). Dietary Polyphenols Promote Growth of the Gut Bacterium Akkermansia muciniphila and Attenuate High-Fat Diet-Induced Metabolic Syndrome. Diabetes.

[117] Akhlaghi M., Ghobadi S., Mohammad Hosseini M., Gholami Z., Mohammadian F. (2018). Flavanols are potential anti-obesity agents, a systematic review and meta-analysis of controlled clinical trials. Nutr. Metab. Cardiovasc. Dis..

[118] Marranzano M., Ray S., Godos J., Galvano F. (2018). Association between dietary flavonoids intake and obesity in a cohort of adults living in the Mediterranean area. Int. J. Food Sci. Nutr..

[119] Hostetler G.L., Ralston R.A., Schwartz S.J. (2017). Flavones: Food Sources, Bioavailability, Metabolism, and Bioactivity. Adv. Nutr..

[120] Hostetler G., Riedl K., Cardenas H., Diosa-Toro M., Arango D., Schwartz S., Doseff A.I. (2012). Flavone deglycosylation increases their anti-inflammatory activity and absorption. Mol. Nutr. Food Res..

[121] Xiao J. (2017). Dietary flavonoid aglycones and their glycosides: Which show better biological significance?. Crit. Rev. Food Sci. Nutr..

[122] Rodriguez-Garcia C., Sanchez-Quesada C., Gaforio J.J. (2019). Dietary Flavonoids as Cancer Chemopreventive Agents: An Updated Review of Human Studies. Antioxidants.

[123] Bhagwat S., Haytowitz D.B., Holden J.M. (2014). USDA Database for the Flavonoid Content of Selected Foods.

[124] Berim A., Gang D.R. (2016). Methoxylated flavones: Occurrence, importance, biosynthesis. Phytochem. Rev..

[125] Ono M., Fujimori K. (2011). Antiadipogenic effect of dietary apigenin through activation of AMPK in 3T3-L1 cells. J. Agric. Food Chem..

[126] Kim M.A., Kang K., Lee H.J., Kim M., Kim C.Y., Nho C.W. (2014). Apigenin isolated from Daphne genkwa Siebold et Zucc. inhibits 3T3-L1 preadipocyte differentiation through a modulation of mitotic clonal expansion. Life Sci..

[127] Gomez-Zorita S., Lasa A., Abendano N., Fernandez-Quintela A., Mosqueda-Solis A., Garcia-Sobreviela M.P., Arbones-Mainar J.M., Portillo M.P. (2017). Phenolic compounds apigenin, hesperidin and kaempferol reduce in vitro lipid accumulation in human adipocytes. J. Transl. Med..

[128] Sun Y.S., Qu W. (2019). Dietary Apigenin promotes lipid catabolism, thermogenesis, and browning in adipose tissues of HFD-Fed mice. Food Chem. Toxicol..

[129] Su T., Huang C., Yang C., Jiang T., Su J., Chen M., Fatima S., Gong R., Hu X., Bian Z. (2019). Apigenin inhibits STAT3/CD36 signaling axis and reduces visceral obesity. Pharmacol. Res..

[130] Feng X., Yu W., Li X., Zhou F., Zhang W., Shen Q., Li J., Zhang C., Shen P. (2017). Apigenin, a modulator of PPARgamma, attenuates HFD-induced NAFLD by regulating hepatocyte lipid metabolism and oxidative stress via Nrf2 activation. Biochem. Pharmacol..

[131] Lv Y., Gao X., Luo Y., Fan W., Shen T., Ding C., Yao M., Song S., Yan L. (2019). Apigenin ameliorates HFD-induced NAFLD through regulation of the XO/NLRP3 pathways. J. Nutr. Biochem..

[132] Feng X., Weng D., Zhou F., Owen Y.D., Qin H., Zhao J., Wen Y., Huang Y., Chen J., Fu H. (2016). Activation of PPARgamma by a Natural Flavonoid Modulator, Apigenin Ameliorates Obesity-Related Inflammation Via Regulation of Macrophage Polarization. EBioMedicine.

[133] Park H.S., Kim S.H., Kim Y.S., Ryu S.Y., Hwang J.T., Yang H.J., Kim G.H., Kwon D.Y., Kim M.S. (2009). Luteolin inhibits adipogenic differentiation by regulating PPARgamma activation. Biofactors.

[134] Kwon E.Y., Kim S.Y., Choi M.S. (2018). Luteolin-Enriched Artichoke Leaf Extract Alleviates the Metabolic Syndrome in Mice with High-Fat Diet-Induced Obesity. Nutrients.

[135] Zhang L., Han Y.J., Zhang X., Wang X., Bao B., Qu W., Liu J. (2016). Luteolin reduces obesity-associated insulin resistance in mice by activating AMPKα1 signalling in adipose tissue macrophages. Diabetologia.

[136] Kwon E.Y., Choi M.S. (2018). Luteolin Targets the Toll-Like Receptor Signaling Pathway in Prevention of Hepatic and Adipocyte Fibrosis and Insulin Resistance in Diet-Induced Obese Mice. Nutrients.

[137] Seo M.-J., Choi H.-S., Lee O.-H., Lee B.-Y. (2013). Baicalein Inhibits Lipid Accumulation through Regulation of MCE and Cell Cycle during 3T3-L1 Adipocyte Differentiation.

[138] Nakao Y., Yoshihara H., Fujimori K. (2016). Suppression of Very Early Stage of Adipogenesis by Baicalein, a Plant-Derived Flavonoid through Reduced Akt-C/EBPalpha-GLUT4 Signaling-Mediated Glucose Uptake in 3T3-L1 Adipocytes. PLoS ONE.

[139] Min W., Wu M., Fang P., Yu M., Shi M., Zhang Z., Bo P. (2018). Effect of Baicalein on GLUT4 Translocation in Adipocytes of Diet-Induced Obese Mice. Cell. Physiol. Biochem..

[140] Nagai S., Matsumoto C., Shibano M., Fujimori K. (2018). Suppression of Fatty Acid and Triglyceride Synthesis by the Flavonoid Orientin through Decrease of C/EBPdelta Expression and Inhibition of PI3K/Akt-FOXO1 Signaling in Adipocytes. Nutrients.

[141] Choi J.H., Yun J.W. (2016). Chrysin induces brown fat-like phenotype and enhances lipid metabolism in 3T3-L1 adipocytes. Nutrition.

[142] Feng X., Qin H., Shi Q., Zhang Y., Zhou F., Wu H., Ding S., Niu Z., Lu Y., Shen P. (2014). Chrysin attenuates inflammation by regulating M1/M2 status via activating PPARgamma. Biochem. Pharmacol..

[143] Hadrich F., Sayadi S. (2018). Apigetrin inhibits adipogenesis in 3T3-L1 cells by downregulating PPARgamma and CEBP-alpha. Lipids Health Dis..

[144] Lee Y.-H., Yang S.-H., Chen S.-L., Pan Y.-F., Liu C.-M., Li M.-W., Chou S.-S., Chou M.-Y., Youn S.-C. (2014). The anti-adipogenic effect of vitexin is via ERK 1/2 MAPK signaling in 3T3-L1 adipocytes. Int. J. Phytomed..

[145] Peng Y., Sun Q., Xu W., He Y., Jin W., Yuan L., Gao R. (2019). Vitexin ameliorates high fat diet-induced obesity in male C57BL/6J mice via the AMPKalpha-mediated pathway. Food Funct..

[146] Na H.Y., Lee B.C. (2019). Scutellaria baicalensis Alleviates Insulin Resistance in Diet-Induced Obese Mice by Modulating Inflammation. Int. J. Mol. Sci..

[147] Shen K., Feng X., Pan H., Zhang F., Xie H., Zheng S. (2017). Baicalin Ameliorates Experimental Liver Cholestasis in Mice by Modulation of Oxidative Stress, Inflammation, and NRF2 Transcription Factor. Oxidative Med. Cell. Longev..

[148] Dai J., Liang K., Zhao S., Jia W., Liu Y., Wu H., Lv J., Cao C., Chen T., Zhuang S. (2018). Chemoproteomics reveals baicalin activates hepatic CPT1 to ameliorate diet-induced obesity and hepatic steatosis. Proc. Natl. Acad. Sci. USA.

[149] Arango D., Morohashi K., Yilmaz A., Kuramochi K., Parihar A., Brahimaj B., Grotewold E., Doseff A.I. (2013). Molecular basis for the action of a dietary flavonoid revealed by the comprehensive identification of apigenin human targets. Proc. Natl. Acad. Sci. USA.

[150] Masson O., Prebois C., Derocq D., Meulle A., Dray C., Daviaud D., Quilliot D., Valet P., Muller C., Liaudet-Coopman E. (2011). Cathepsin-D, a key protease in breast cancer, is up-regulated in obese mouse and human adipose tissue, and controls adipogenesis. PLoS ONE.

[151] Eguchi A., Feldstein A.E. (2013). Lysosomal Cathepsin D contributes to cell death during adipocyte hypertrophy. Adipocyte.

[152] Ando C., Takahashi N., Hirai S., Nishimura K., Lin S., Uemura T., Goto T., Yu R., Nakagami J., Murakami S. (2009). Luteolin, a food-derived flavonoid, suppresses adipocyte-dependent activation of macrophages by inhibiting JNK activation. FEBS Lett..

[153] Gerhardt C.C., Romero I.A., Cancello R., Camoin L., Strosberg A.D. (2001). Chemokines control fat accumulation and leptin secretion by cultured human adipocytes. Mol. Cell. Endocrinol..

[154] Kaplan J.L., Marshall M.A., McSkimming C.C., Harmon D.B., Garmey J.C., Oldham S.N., Hallowell P., McNamara C.A. (2015). Adipocyte progenitor cells initiate monocyte chemoattractant protein-1-mediated macrophage accumulation in visceral adipose tissue. Mol. Metab..

[155] Parekh N., Chandran U., Bandera E.V. (2012). Obesity in cancer survival. Annu. Rev. Nutr..

[156] Makowski L., Zhou C., Zhong Y., Kuan P.F., Fan C., Sampey B.P., Difurio M., Bae-Jump V.L. (2014). Obesity increases tumor aggressiveness in a genetically engineered mouse model of serous ovarian cancer. Gynecol. Oncol..

[157] Dai Z., Xu Y.C., Niu L. (2007). Obesity and colorectal cancer risk: A meta-analysis of cohort studies. World J. Gastroenterol..

[158] Nieman K.M., Romero I.L., Van Houten B., Lengyel E. (2013). Adipose tissue and adipocytes support tumorigenesis and metastasis. Biochim. Biophys. Acta.

[159] Wang Y.Y., Attane C., Milhas D., Dirat B., Dauvillier S., Guerard A., Gilhodes J., Lazar I., Alet N., Laurent V. (2017). Mammary adipocytes stimulate breast cancer invasion through metabolic remodeling of tumor cells. JCI Insight.

[160] Iyengar P., Combs T.P., Shah S.J., Gouon-Evans V., Pollard J.W., Albanese C., Flanagan L., Tenniswood M.P., Guha C., Lisanti M.P. (2003). Adipocyte-secreted factors synergistically promote mammary tumorigenesis through induction of anti-apoptotic transcriptional programs and proto-oncogene stabilization. Oncogene.

[161] Park J., Morley T.S., Kim M., Clegg D.J., Scherer P.E. (2014). Obesity and cancer--mechanisms underlying tumour progression and recurrence. Nat. Rev. Endocrinol..

[162] Park J., Euhus D.M., Scherer P.E. (2011). Paracrine and endocrine effects of adipose tissue on cancer development and progression. Endocr. Rev..

[163] Chen Y., He Y., Wang X., Lu F., Gao J. (2019). Adiposederived mesenchymal stem cells exhibit tumor tropism and promote tumorsphere formation of breast cancer cells. Oncol. Rep..

[164] Goto H., Shimono Y., Funakoshi Y., Imamura Y., Toyoda M., Kiyota N., Kono S., Takao S., Mukohara T., Minami H. (2019). Adipose-derived stem cells enhance human breast cancer growth and cancer stem cell-like properties through adipsin. Oncogene.

[165] Gonzalez-Perez R.R., Xu Y., Guo S., Watters A., Zhou W., Leibovich S.J. (2010). Leptin upregulates VEGF in breast cancer via canonic and non-canonical signalling pathways and NFkappaB/HIF-1alpha activation. Cell Signal..

[166] Zheng Q., Banaszak L., Fracci S., Basali D., Dunlap S.M., Hursting S.D., Rich J.N., Hjlemeland A.B., Vasanji A., Berger N.A. (2013). Leptin receptor maintains cancer stem-like properties in triple negative breast cancer cells. Endocr. Relat. Cancer.

[167] Chakraborty D., Benham V., Bullard B., Kearney T., Hsia H.C., Gibbon D., Demireva E.Y., Lunt S.Y., Bernard J.J. (2017). Fibroblast growth factor receptor is a mechanistic link between visceral adiposity and cancer. Oncogene.

[168] Harris A.L. (2002). Hypoxia—A key regulatory factor in tumour growth. Nat. Rev. Cancer.

[169] Cao Y. (2010). Adipose tissue angiogenesis as a therapeutic target for obesity and metabolic diseases. Nat. Rev. Drug Discov..

[170] Bu D., Crewe C., Kusminski C.M., Gordillo R., Ghaben A.L., Kim M., Park J., Deng H., Xiong W., Liu X.Z. (2019). Human endotrophin as a driver of malignant tumor growth. JCI Insight.

[171] Iyengar P., Espina V., Williams T.W., Lin Y., Berry D., Jelicks L.A., Lee H., Temple K., Graves R., Pollard J. (2005). Adipocyte-derived collagen VI affects early mammary tumor progression in vivo, demonstrating a critical interaction in the tumor/stroma microenvironment. J. Clin. Investig..

[172] Park J., Scherer P.E. (2012). Endotrophin—A novel factor linking obesity with aggressive tumor growth. Oncotarget.

[173] Dirat B., Bochet L., Dabek M., Daviaud D., Dauvillier S., Majed B., Wang Y.Y., Meulle A., Salles B., Le Gonidec S. (2011). Cancer-associated adipocytes exhibit an activated phenotype and contribute to breast cancer invasion. Cancer Res..

[174] Madak-Erdogan Z., Band S., Zhao Y.C., Smith B.P., Kulkoyluoglu-Cotul E., Zuo Q., Santaliz Casiano A., Wrobel K., Rossi G., Smith R.L. (2019). Free Fatty Acids Rewire Cancer Metabolism in Obesity-Associated Breast Cancer via Estrogen Receptor and mTOR Signaling. Cancer Res..

[175] Park E.J., Lee J.H., Yu G.Y., He G., Ali S.R., Holzer R.G., Osterreicher C.H., Takahashi H., Karin M. (2010). Dietary and genetic obesity promote liver inflammation and tumorigenesis by enhancing IL-6 and TNF expression. Cell.

[176] Subbaramaiah K., Howe L.R., Bhardwaj P., Du B., Gravaghi C., Yantiss R.K., Zhou X.K., Blaho V.A., Hla T., Yang P. (2011). Obesity is associated with inflammation and elevated aromatase expression in the mouse mammary gland. Cancer Prev. Res. (Phila).

[177] Pollard J.W. (2004). Tumour-educated macrophages promote tumour progression and metastasis. Nat. Rev. Cancer.

[178] Bougaret L., Delort L., Billard H., Le Huede C., Boby C., De la Foye A., Rossary A., Mojallal A., Damour O., Auxenfans C. (2018). Adipocyte/breast cancer cell crosstalk in obesity interferes with the anti-proliferative efficacy of tamoxifen. PLoS ONE.

[179] Vargo M.A., Voss O.H., Poustka F., Cardounel A.J., Grotewold E., Doseff A.I. (2006). Apigenin-induced-apoptosis is mediated by the activation of PKCdelta and caspases in leukemia cells. Biochem. Pharmacol..

[180] Arango D., Parihar A., Villamena F.A., Wang L., Freitas M.A., Grotewold E., Doseff A.I. (2012). Apigenin induces DNA damage through the PKCdelta-dependent activation of ATM and H2AX causing down-regulation of genes involved in cell cycle control and DNA repair. Biochem. Pharmacol..

[181] Kang K.A., Piao M.J., Hyun Y.J., Zhen A.X., Cho S.J., Ahn M.J., Yi J.M., Hyun J.W. (2019). Luteolin promotes apoptotic cell death via upregulation of Nrf2 expression by DNA demethylase and the interaction of Nrf2 with p53 in human colon cancer cells. Exp. Mol. Med..

[182] Shukla S., Gupta S. (2009). Apigenin suppresses insulin-like growth factor I receptor signaling in human prostate cancer: An in vitro and in vivo study. Mol. Carcinog..

[183] Li Y.W., Xu J., Zhu G.Y., Huang Z.J., Lu Y., Li X.Q., Wang N., Zhang F.X. (2018). Apigenin suppresses the stem cell-like properties of triple-negative breast cancer cells by inhibiting YAP/TAZ activity. Cell Death Discov..

[184] Koh S.Y., Moon J.Y., Unno T., Cho S.K. (2019). Baicalein Suppresses Stem Cell-Like Characteristics in Radio- and Chemoresistant MDA-MB-231 Human Breast Cancer Cells through Up-Regulation of IFIT2. Nutrients.

[185] Arango D., Diosa-Toro M., Rojas-Hernandez L.S., Cooperstone J.L., Schwartz S.J., Mo X., Jiang J., Schmittgen T.D., Doseff A.I. (2015). Dietary apigenin reduces LPS-induced expression of miR-155 restoring immune balance during inflammation. Mol. Nutr. Food Res..

[186] Cardenas H., Arango D., Nicholas C., Duarte S., Nuovo G.J., He W., Voss O.H., Gonzalez-Mejia M.E., Guttridge D.C., Grotewold E. (2016). Dietary Apigenin Exerts Immune-Regulatory Activity in Vivo by Reducing NF-kappaB Activity, Halting Leukocyte Infiltration and Restoring Normal Metabolic Function. Int. J. Mol. Sci..

[187] Nicholas C., Batra S., Vargo M.A., Voss O.H., Gavrilin M.A., Wewers M.D., Guttridge D.C., Grotewold E., Doseff A.I. (2007). Apigenin blocks lipopolysaccharide-induced lethality in vivo and proinflammatory cytokines expression by inactivating NF-κB through the suppression of p65 phosphorylation. J. Immunol..

[188] Bauer D., Redmon N., Mazzio E., Soliman K.F. (2017). Apigenin inhibits TNFalpha/IL-1alpha-induced CCL2 release through IKBK-epsilon signaling in MDA-MB-231 human breast cancer cells. PLoS ONE.

[189] Choi H.J., Choi H.J., Chung T.W., Ha K.T. (2016). Luteolin inhibits recruitment of monocytes and migration of Lewis lung carcinoma cells by suppressing chemokine (C-C motif) ligand 2 expression in tumor-associated macrophage. Biochem. Biophys. Res. Commun..

[190] Fang B., Chen X., Wu M., Kong H., Chu G., Zhou Z., Zhang C., Chen B. (2018). Luteolin inhibits angiogenesis of the M2like TAMs via the downregulation of hypoxia inducible factor1alpha and the STAT3 signalling pathway under hypoxia. Mol. Med. Rep..

[191] Brown E.M., Sadarangani M., Finlay B.B. (2013). The role of the immune system in governing host-microbe interactions in the intestine. Nat. Immunol..

[192] Zierer J., Jackson M.A., Kastenmuller G., Mangino M., Long T., Telenti A., Mohney R.P., Small K.S., Bell J.T., Steves C.J. (2018). The fecal metabolome as a functional readout of the gut microbiome. Nat. Genet..

[193] David L.A., Maurice C.F., Carmody R.N., Gootenberg D.B., Button J.E., Wolfe B.E., Ling A.V., Devlin A.S., Varma Y., Fischbach M.A. (2014). Diet rapidly and reproducibly alters the human gut microbiome. Nature.

[194] Turnbaugh P.J., Hamady M., Yatsunenko T., Cantarel B.L., Duncan A., Ley R.E., Sogin M.L., Jones W.J., Roe B.A., Affourtit J.P. (2009). A core gut microbiome in obese and lean twins. Nature.

[195] Shen W., Gaskins H.R., McIntosh M.K. (2014). Influence of dietary fat on intestinal microbes, inflammation, barrier function and metabolic outcomes. J. Nutr. Biochem..

[196] Xiao S., Zhao L. (2014). Gut microbiota-based translational biomarkers to prevent metabolic syndrome via nutritional modulation. FEMS Microbiol. Ecol..

[197] Backhed F., Manchester J.K., Semenkovich C.F., Gordon J.I. (2007). Mechanisms underlying the resistance to diet-induced obesity in germ-free mice. Proc. Natl. Acad. Sci. USA.

[198] Ley R.E., Backhed F., Turnbaugh P., Lozupone C.A., Knight R.D., Gordon J.I. (2005). Obesity alters gut microbial ecology. Proc. Natl. Acad. Sci. USA.

[199] Hildebrandt M.A., Hoffmann C., Sherrill-Mix S.A., Keilbaugh S.A., Hamady M., Chen Y.Y., Knight R., Ahima R.S., Bushman F., Wu G.D. (2009). High-fat diet determines the composition of the murine gut microbiome independently of obesity. Gastroenterology.

[200] Turnbaugh P.J., Ley R.E., Mahowald M.A., Magrini V., Mardis E.R., Gordon J.I. (2006). An obesity-associated gut microbiome with increased capacity for energy harvest. Nature.

[201] Ley R.E., Turnbaugh P.J., Klein S., Gordon J.I. (2006). Human gut microbes associated with obesity. Nature.

[202] Kim K.A., Gu W., Lee I.A., Joh E.H., Kim D.H. (2012). High fat diet-induced gut microbiota exacerbates inflammation and obesity in mice via the TLR4 signaling pathway. PLoS ONE.

[203] Luck H., Tsai S., Chung J., Clemente-Casares X., Ghazarian M., Revelo X.S., Lei H., Luk C.T., Shi S.Y., Surendra A. (2015). Regulation of obesity-related insulin resistance with gut anti-inflammatory agents. Cell Metab..

[204] Winer D.A., Luck H., Tsai S., Winer S. (2016). The Intestinal Immune System in Obesity and Insulin Resistance. Cell Metab..

[205] Kawano Y., Nakae J., Watanabe N., Kikuchi T., Tateya S., Tamori Y., Kaneko M., Abe T., Onodera M., Itoh H. (2016). Colonic Pro-inflammatory Macrophages Cause Insulin Resistance in an Intestinal Ccl2/Ccr2-Dependent Manner. Cell Metab..

[206] Seregin S.S., Golovchenko N., Schaf B., Chen J., Pudlo N.A., Mitchell J., Baxter N.T., Zhao L., Schloss P.D., Martens E.C. (2017). NLRP6 Protects Il10(-/-) Mice from Colitis by Limiting Colonization of Akkermansia muciniphila. Cell Rep..

[207] Xiao L., Chen B., Feng D., Yang T., Li T., Chen J. (2019). TLR4 May Be Involved in the Regulation of Colonic Mucosal Microbiota by Vitamin A. Front. Microbiol..

[208] Ding S., Chi M.M., Scull B.P., Rigby R., Schwerbrock N.M., Magness S., Jobin C., Lund P.K. (2010). High-fat diet: Bacteria interactions promote intestinal inflammation which precedes and correlates with obesity and insulin resistance in mouse. PLoS ONE.

[209] Cani P.D., Possemiers S., Van de Wiele T., Guiot Y., Everard A., Rottier O., Geurts L., Naslain D., Neyrinck A., Lambert D.M. (2009). Changes in gut microbiota control inflammation in obese mice through a mechanism involving GLP-2-driven improvement of gut permeability. Gut.

[210] Schulz M.D., Atay C., Heringer J., Romrig F.K., Schwitalla S., Aydin B., Ziegler P.K., Varga J., Reindl W., Pommerenke C. (2014). High-fat-diet-mediated dysbiosis promotes intestinal carcinogenesis independently of obesity. Nature.

[211] Poutahidis T., Varian B.J., Levkovich T., Lakritz J.R., Mirabal S., Kwok C., Ibrahim Y.M., Kearney S.M., Chatzigiagkos A., Alm E.J. (2015). Dietary microbes modulate transgenerational cancer risk. Cancer Res..

[212] Routy B., Le Chatelier E., Derosa L., Duong C.P.M., Alou M.T., Daillere R., Fluckiger A., Messaoudene M., Rauber C., Roberti M.P. (2018). Gut microbiome influences efficacy of PD-1-based immunotherapy against epithelial tumors. Science.

[213] Laparra J.M., Sanz Y. (2010). Interactions of gut microbiota with functional food components and nutraceuticals. Pharmacol. Res..

[214] Ahn-Jarvis J.H., Parihar A., Doseff A.I. (2019). Dietary Flavonoids for Immunoregulation and Cancer: Food Design for Targeting Disease. Antioxidants.

[215] Aura A.-M. (2008). Microbial metabolism of dietary phenolic compounds in the colon. Phytochem. Rev..

[216] Kawabata K., Yoshioka Y., Terao J. (2019). Role of Intestinal Microbiota in the Bioavailability and Physiological Functions of Dietary Polyphenols. Molecules.

[217] Comalada M., Camuesco D., Sierra S., Ballester I., Xaus J., Gálvez J., Zarzuelo A. (2005). In vivo quercitrin anti-inflammatory effect involves release of quercetin, which inhibits inflammation through down-regulation of the NF-κB pathway. Eur. J. Immunol..

[218] Lee H.C., Jenner A.M., Low C.S., Lee Y.K. (2006). Effect of tea phenolics and their aromatic fecal bacterial metabolites on intestinal microbiota. Res. Microbiol..

[219] Collins B., Hoffman J., Martinez K., Grace M., Lila M.A., Cockrell C., Nadimpalli A., Chang E., Chuang C.C., Zhong W. (2016). A polyphenol-rich fraction obtained from table grapes decreases adiposity, insulin resistance and markers of inflammation and impacts gut microbiota in high-fat-fed mice. J. Nutr. Biochem..

[220] Zhao L., Zhang Q., Ma W., Tian F., Shen H., Zhou M. (2017). A combination of quercetin and resveratrol reduces obesity in high-fat diet-fed rats by modulation of gut microbiota. Food Funct..

[221] Etxeberria U., Arias N., Boque N., Macarulla M.T., Portillo M.P., Martinez J.A., Milagro F.I. (2015). Reshaping faecal gut microbiota composition by the intake of trans-resveratrol and quercetin in high-fat sucrose diet-fed rats. J. Nutr. Biochem..

[222] Porras D., Nistal E., Martinez-Florez S., Pisonero-Vaquero S., Olcoz J.L., Jover R., Gonzalez-Gallego J., Garcia-Mediavilla M.V., Sanchez-Campos S. (2017). Protective effect of quercetin on high-fat diet-induced non-alcoholic fatty liver disease in mice is mediated by modulating intestinal microbiota imbalance and related gut-liver axis activation. Free Radic. Biol. Med..

[223] Anhe F.F., Roy D., Pilon G., Dudonne S., Matamoros S., Varin T.V., Garofalo C., Moine Q., Desjardins Y., Levy E. (2015). A polyphenol-rich cranberry extract protects from diet-induced obesity, insulin resistance and intestinal inflammation in association with increased Akkermansia spp. population in the gut microbiota of mice. Gut.

[224] den Besten G., Bleeker A., Gerding A., van Eunen K., Havinga R., van Dijk T.H., Oosterveer M.H., Jonker J.W., Groen A.K., Reijngoud D.J. (2015). Short-Chain Fatty Acids Protect Against High-Fat Diet-Induced Obesity via a PPARgamma-Dependent Switch from Lipogenesis to Fat Oxidation. Diabetes.

[225] Noratto G.D., Garcia-Mazcorro J.F., Markel M., Martino H.S., Minamoto Y., Steiner J.M., Byrne D., Suchodolski J.S., Mertens-Talcott S.U. (2014). Carbohydrate-Free Peach (*Prunus persica*) and Plum (*Prunus salicina*) [corrected] Juice Affects Fecal Microbial Ecology in an Obese Animal Model. PLoS ONE.

[226] Ghimire S., Wongkuna S., Sankaranarayanan R., Ryan E.P., Bhat G.J., Scaria J. (2020). Rice Bran and Quercetin Produce a Positive Synergistic Effect on Human Gut Microbiota, Elevate the Level of Propionate, and Reduce the Population of *Enterobacteriaceae* family when Determined using a Bioreactor Model. bioRxiv.

[227] Jaimes J.D., Jarosova V., Vesely O., Mekadim C., Mrazek J., Marsik P., Killer J., Smejkal K., Kloucek P., Havlik J. (2019). Effect of Selected Stilbenoids on Human Fecal Microbiota. Molecules.

[228] Nakatsu C.H., Armstrong A., Clavijo A.P., Martin B.R., Barnes S., Weaver C.M. (2014). Fecal bacterial community changes associated with isoflavone metabolites in postmenopausal women after soy bar consumption. PLoS ONE.

[229] Radulovic K., Normand S., Rehman A., Delanoye-Crespin A., Chatagnon J., Delacre M., Waldschmitt N., Poulin L.F., Iovanna J., Ryffel B. (2018). A dietary flavone confers communicable protection against colitis through NLRP6 signaling independently of inflammasome activation. Mucosal Immunol..

[230] Wang M., Firrman J., Zhang L., Arango-Argoty G., Tomasula P., Liu L., Xiao W., Yam K. (2017). Apigenin Impacts the Growth of the Gut Microbiota and Alters the Gene Expression of Enterococcus. Molecules.

